# Do anti-malarials in Africa meet quality standards? The market penetration of non quality-assured artemisinin combination therapy in eight African countries

**DOI:** 10.1186/s12936-017-1818-8

**Published:** 2017-05-25

**Authors:** ACTwatch Group, Louis Akulayi, Angela Alum, Andrew Andrada, Julie Archer, Ekundayo D. Arogundade, Erick Auko, Abdul R. Badru, Katie Bates, Paul Bouanchaud, Meghan Bruce, Katia Bruxvoort, Peter Buyungo, Angela Camilleri, Emily D. Carter, Steven Chapman, Nikki Charman, Desmond Chavasse, Robyn Cyr, Kevin Duff, Keith Esch, Illah Evance, Anna Fulton, Hellen Gataaka, Gylsain Guedegbe, Tarryn Haslam, Emily Harris, Christine Hong, Catharine Hurley, Whitney Isenhower, Enid Kaabunga, Baraka D. Kaaya, Esther Kabui, Beth Kangwana, Lason Kapata, Henry Kaula, Gloria Kigo, Irene Kyomuhangi, Aliza Lailari, Sandra LeFevre, Megan Littrell, Greta Martin, Daniel Michael, Erik Monroe, Godefroid Mpanya, Felton Mpasela, Felix Mulama, Anne Musuva, Julius Ngigi, Edward Ngoma, Marjorie Norman, Bernard Nyauchi, Kathryn A. O’Connell, Carolyne Ochieng, Edna Ogada, Linda Ongwenyi, Ricki Orford, Saysana Phanalasy, Stephen Poyer, Justin Rahariniaina, Jacky Raharinjatovo, Lanto Razafindralambo, Solofo Razakamiadana, Christina Riley, John Rodgers, Andria Rusk, Tanya Shewchuk, Simon Sensalire, Julianna Smith, Phok Sochea, Tsione Solomon, Raymond Sudoi, Martine Esther Tassiba, Katherine Thanel, Rachel Thompson, Mitsuru Toda, Chinazo Ujuju, Marie-Alix Valensi, Vamsi Vasireddy, Cynthia B. Whitman, Cyprien Zinsou, Paul N. Newton, Kara Hanson, Catherine Goodman

**Affiliations:** 1 Population Services International, 1120 19th St NW Suite 600, Washington, DC 20036 USA; 2 Centre for Tropical Medicine and Global Health, Nuffield Department of Clinical Medicine, University of Oxford, Oxford, UK; 3 London School of Hygiene and Tropical Medicine, 15-17 Tavistock Place, London, WCH 9SH UK

**Keywords:** ACT, Anti-malarial, Medicine quality, Regulation

## Abstract

**Background:**

Quality of artemisinin-based combination therapy (ACT) is important for ensuring malaria parasite clearance and protecting the efficacy of artemisinin-based therapies. The extent to which non quality-assured ACT (non-QAACT), or those not granted global regulatory approval, are available and used to treat malaria in endemic countries is poorly documented. This paper uses national and sub-national medicine outlet surveys conducted in eight study countries (Benin, Kinshasa and Kantanga [Democratic Republic of the Congo, DRC], Kenya, Madagascar, Nigeria, Tanzania, Uganda and Zambia) between 2009 and 2015 to describe the non-QAACT market and to document trends in availability and distribution of non-QAACT in the public and private sector.

**Results:**

In 2014/15, non-QAACT were most commonly available in Kinshasa (83%), followed by Katanga (53%), Nigeria (48%), Kenya (42%), and Uganda (33%). Non-QAACT accounted for 20% of the market share in the private sector in Kenya, followed by Benin and Uganda (19%), Nigeria (12%) and Zambia (8%); this figure was 27% in Katanga and 40% in Kinshasa. Public sector non-QAACT availability and distribution was much lower, with the exception of Zambia (availability, 85%; market share, 32%). Diverse generics and formulations were available, but non-QAACT were most commonly artemether–lumefantrine (AL) or dihydroartemisinin-piperaquine (DHA PPQ), in tablet formulation, imported, and distributed in urban areas at either pharmacies or drug stores. The number of unique manufacturers supplying non-QAACT to each country ranged from 9 in Uganda to 92 in Nigeria.

**Conclusions:**

Addressing the availability and distribution of non-QAACT will require effective private sector engagement and evidence-based strategies to address provider and consumer demand for these products. Given the variation in non-QAACT markets observed across the eight study countries, active efforts to limit registration, importation and distribution of non-QAACT must be tailored to the country context, and will involve addressing complex and challenging aspects of medicine registration, private sector pharmaceutical regulation, local manufacturing and drug importation. These efforts may be critical not only to patient health and safety, but also to effective malaria control and protection of artemisinin drug efficacy in the face of spreading resistance.

**Electronic supplementary material:**

The online version of this article (doi:10.1186/s12936-017-1818-8) contains supplementary material, which is available to authorized users.

## Background

The consequences and dangers of poor quality anti-malarials are extensive. They contributed to an estimated 91,577–154,736 deaths among African children under the age of five alone in 2013 [1]. Poor quality anti-malarials containing sub-therapeutic doses of the active pharmaceutical ingredient (API) may be ineffective at clearing malaria parasites, leading to prolonged illness or even patient death. Additional undisclosed ingredients could pose an independent threat to consumer health or could interact adversely with a patient’s existing medication regimen. Aside from causing morbidity and mortality, poor quality artemisinin combination therapy (ACT) medicines—the recommended first-line treatment for uncomplicated malaria in sub-Saharan Africa (SSA)—waste consumer money and may decrease confidence among consumers and providers in the efficacy of ACT. Poor quality anti-malarials are also critical to effective malaria control and protection of artemisinin and partner drug efficacy given their use can promote drug resistance [2–5]. In particular, the emergence of artemisinin resistance, likely resulting from decades of sub-therapeutic monotherapy and substandard artemisinin derivative consumption [6], has prompted increased attention to anti-malarial medicine quality in recent years. Medicine quality is one of the many threats to appropriate and effective malaria case management, along with other factors such as lack of or incorrect parasitological diagnosis, use of non-artemisinin therapies, insufficient access to quality-assured ACT (QAACT), and poor medication adherence by consumers [3, 4]. Nonetheless, the presence of potentially poor quality anti-malarials in the market is clearly a key cause for concern in the fight against malaria.

Poor quality anti-malarials include substandard and falsified medicines. The term ‘substandard’ refers to medicines that may not contain the indicated amount of API and/or may have poor dissolution of the API [5, 7]. Substandard drugs include both poorly manufactured medicines, and degraded medicines whose contents and therapeutic value were negatively affected during storage or distribution by extreme temperature or time. The compromised quality of these substandard and degraded drugs is a result of failures in quality monitoring along production and supply chains. In contrast, falsified anti-malarials are produced fraudulently and labeling contains false claims on content and origin. These medicines may contain little or none of the claimed API, and may include incorrect, unstated substances [7].

In SSA, where 90% of the global malaria mortality burden is concentrated [8], a major barrier to addressing poor quality anti-malarials is gauging the extent of the problem. A number of anti-malarial drug quality studies have been conducted in recent years [9]. These studies involve sampling and testing active ingredients and have been useful in providing some indication of the extent to which poor quality anti-malarials are available. In recent ACT drug quality studies in SSA, between 0.3 and 66.7% of drugs studied were found to be outside the acceptable API range [3, 10–16]. In a review of falsified and substandard medicines, eight prevalence estimates from sub-Saharan Africa ranged from 12.2 to 48%, with a median of 34.5% [17]. However, such anti-malarial quality studies tend to be conducted on a sub-national scale and use convenience sampling, small sample sizes, and variable techniques for chemical quality analysis  [3, 5, 7, 18, 19]. While this evidence on poor quality anti-malarials has been aggregated in databases such as the WorldWide Anti-malarial Resistance Network’s (WWARN) Anti-malarial Quality map [9], the variation in individual study methodologies makes identifying trends and generalization beyond study settings challenging. Thus, it has been difficult to determine the scale of the problem in high malaria burden countries due to the lack of available standardized and comprehensive data with which to characterize the market for poor quality medicines [4]. As the majority of anti-malarials in SSA countries are distributed by the private sector, understanding this market and improving private provider practices related to quality-assurance is essential [20].

**Fig. 1:**
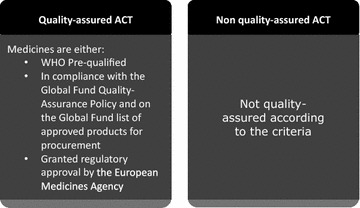
Defining QAACT and non-QAACT

At the global level, one strategy for ensuring availability and use of quality anti-malarials is medicine prequalification. Prequalification programs are designed to identify medicines that are manufactured according to quality standards yielding safe and efficacious medicines. Approval through these mechanisms varies, but typically requires the manufacturer to submit an application, documentation of the chemical and pharmaceutical properties of the product, bioequivalence tests (if generic), package labeling, proof of in-country registration, and a record of facilities’ good manufacturing processes, for review by a panel of experts [21–25]. Depending on the reviewer body, applicants may also be required to pay a processing fee [23–26]. Anti-malarials designated as pre-qualified or granted regulatory approval by global authorities such as the World Health Organization (WHO), may be considered “quality-assured” (Fig. 1). This quality designation has been leveraged to promote private sector distribution of quality anti-malarials by the Global Fund’s Private Sector Copayment Mechanism, first piloted in 2010–2011 as the Affordable Medicines Facility-malaria (AMFm), by facilitating first-line buyer access to affordable quality-assured medicines [27].

**Fig. 2:**
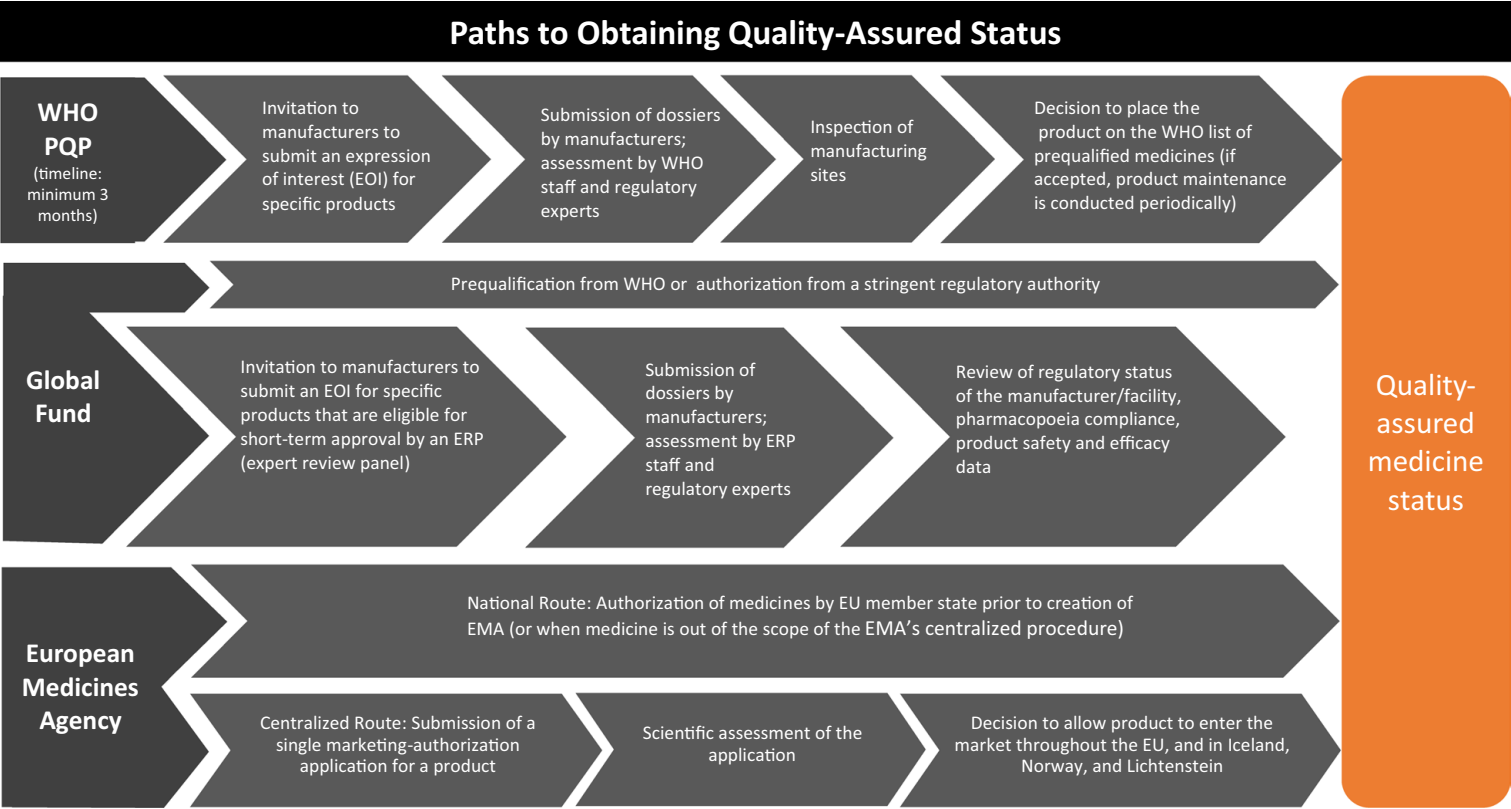
Paths to obtaining quality assured status

Malaria-endemic countries in SSA have limited resources at the country level for evaluating the safety and efficacy of anti-malarials and implementing regulatory processes [21, 23]. As such, global medicine pre-qualification is an essential tool for ensuring that medicines circulating in the global marketplace are of high quality. The World Health Organization’s Prequalification Program (WHO PQP), developed in 2001, serves as a global regulator. Using the Good Manufacturing Practices (GMP) defined by the World Health Assembly in the 1960s as a foundation, the WHO PQP aims to identify medicines that meet “unified standards for quality, safety, and efficacy”. The programme employs a rigorous review and approval process to qualify medicines and products for procurement by U.N. agencies. In doing so, the WHO PQP seeks to standardize international drug quality and ensure access to priority essential medicines [28]. Other entities, such as the Global Fund and the European Medicines Agency (EMA) have developed their own, sometimes complementary, processes to approve medicines for global market entry [29, 30] (Fig. 2). WHO PQP status is often viewed as a global standard for drug quality, especially since organizations like the Global Fund use the WHO PQP list as a base to form approved product procurement lists.

GMP and quality-assurance status granted by regulatory authorities do not necessarily preclude manufacturing quality failures or prevent conditions or practices that may lead to drug degradation over time. Moreover, medicines that have not been granted pre-qualification status or regulatory approval may be safe and efficacious. Nonetheless, quality-assurance status has been associated with high quality medicines in field drug quality studies [31]. A nationally representative survey of over 1700 anti-malarials in Tanzania’s private sector found that ACT samples lacking WHO prequalification were 25 times more likely to be of poor quality than those with WHO prequalification status [14]. When adjusting for date of expiry among ACT, lack of WHO prequalification was the strongest predictor of poor quality in a multivariate analysis. The results of this study highlight that quality-assurance status can serve as an important indicator of ACT drug quality.

This paper uses data from 29 malaria medicine outlet surveys conducted under the ACTwatch project between 2009 and 2015 in eight country contexts (Benin, Kinshasa and Katanga in the Democratic Republic of Congo [DRC], Kenya, Madagascar, Nigeria, Tanzania, Uganda, and Zambia) to examine the extent to which non-QAACT are available and distributed to consumers. ACT were classified as quality-assured or non quality-assured according to approval status by the WHO pre-qualification programme, the Global Fund, or the EMA (see Fig. 1 for quality classification criteria and Fig. 2 for the drug approval processes by entity). During the study time period, four of the study countries (Kenya, Nigeria, Uganda and Tanzania) saw growth in private sector availability and distribution of QAACT due to the Private Sector Copayment Mechanism [27]. Given this evidence, this study also examines availability and market share for non-QAACT in contexts with and without large-scale private sector market interventions to improve access to QAACT.

## Methods

ACTwatch was launched in 2008 with the goal to generate timely, relevant and high quality evidence about anti-malarial markets for policy makers, donors and implementing organizations. Both supply and demand sides of the anti-malarial market were addressed, through outlet and household surveys, supply chain analysis, key informant interviews and exit interviews for consumers of anti-malarial-stocking outlets. As of 2016, ACTwatch had gathered data from 12 malaria endemic countries in sub-Saharan Africa and the Greater Mekong sub-Region. Detailed ACTwatch project and methodological information have been published elsewhere  [32, 33].

### Design and sampling

ACTwatch outlet surveys were nationally-representative (with the exception of the sub-national surveys in the DRC), cross-sectional quantitative surveys conducted among a sample of outlets stocking anti-malarials. Surveys were repeated over time to inform, monitor and evaluate policies and strategies designed to improve access and use of malaria diagnostics and first-line treatments.

All categories of outlets with the potential to stock anti-malarials in both the public/not-for-profit and private-for-profit sector were included in the study. In the public/not-for-profit sector (hereafter referred to as simply the ‘public sector’), this included government and non-government not-for-profit health facilities (hospitals, centers, clinics and posts) and community health workers. Outlets sampled in the private sector included private for-profit health facilities (hospitals, centers and clinics), pharmacies, drug stores (registered/regulated and unregistered/unregulated), general retailers selling fast-moving consumer goods, and itinerant drug vendors (mobile vendors without a fixed service delivery point).

Lists of all potentially eligible outlets were not routinely available and therefore a cluster sampling approach with an outlet census was used to identify outlets for inclusion. Clusters were administrative units ideally with a typical size of 10,000–15,000 inhabitants, and were selected using probability proportional to population size (PPS) sampling. Within each selected cluster all outlets with the potential to provide anti-malarials to consumers were screened for eligibility. Outlets were eligible for an anti-malarial product audit if they had one or more anti-malarials in stock on the survey day.

Boundaries for the outlet census were typically extended to higher administrative units to cover a larger area for the census of public health facilities and pharmacies, in order to over-sample these relatively uncommon but important outlet types.

Each survey was stratified to deliver estimates for relevant research domains: all countries had urban and rural stratification, with the exception of Nigeria for which six geopolitical zones were used as research domains. Each study round was powered to detect a minimum of a 20% point change in availability of QAACT among anti-malarial stocking outlets between each round and within each domain at the 5% significance level with 80% power. The number of study clusters was calculated for each research domain based on the required number of anti-malarial stocking outlets and assumptions about the number of anti-malarial stocking outlets per cluster. Sample size requirements for follow-up surveys were calculated using information from previous survey rounds including anti-malarial and QAACT availability, outlet density per cluster, and design effect.

Data collection periods varied by country and over time but were typically implemented during the peak malaria transmission season for each country and lasted 6–8 weeks. Efforts were made to ensure surveys were implemented over similar time points across the survey rounds.

### Training and fieldwork

Interviewer training consisted of standardized classroom presentations and exercises as well as a field exercise. Exams administered during training were used to select data collectors, supervisors, and quality-controllers. Additional training was provided for supervisors and quality-controllers focused on field monitoring, verification visits, and census procedures. Data collection teams were provided with a list of selected clusters and official maps that illustrated their administrative boundaries. In each selected cluster, fieldworkers conducted a full enumeration of all outlets that had the potential to provide anti-malarials. This included enumeration of outlets with a physical location, as well as identification of community health workers and itinerant drug vendors using local informants and snowball sampling. The primary provider/owner of each outlet was invited to participate in the study and screening questions were administered to assess anti-malarial availability. Interviews were conducted in local language(s) and questionnaires underwent forward and backward translation from English to the local language. Quality control measures implemented during data collection included questionnaire review by supervisors and a minimum of 10% of outlets were back-checked.

### Measures

The outlet survey questionnaire included an audit of all available anti-malarials. Providers were asked to show the interviewer all anti-malarials currently available. A product audit sheet captured information for each unique anti-malarial product in the outlet, including formulation, brand name, active ingredient(s) and strength(s), package size, manufacturer and country of manufacture. Providers were asked to report the retail and wholesale cost for each medicine, as well as the amount distributed to individual consumers in the last week. All surveys were paper-based with the exception of Madagascar in 2015 and Uganda in 2015, where data were collected using Android phones and forms created using DroidDB (^©^ SYWARE, Inc., Cambridge, MA, USA).

### Protection of human subjects

The outlet survey protocols received ethical approval from national ethical approval boards within each country and for each survey round. Ethical clearance for the last survey round was as follows: DRC, ESP/CE/096/2015; Kenya, KNH-ERC/A/360; Madagascar, 090-MSANP/CE; Nigeria, NHREC/01/01/2007-09/07/2015; Tanzania, NIMR/HQ/R.8a/Vol. IX/1840; Uganda, 2008-057; Zambia, IRB00001131. Provider interviews and product audits were completed only after administration of a standard informed consent form and provider consent to participate in the study. Providers had the option to end the interview at any point during the study. Standard measures were employed to maintain provider confidentiality and anonymity, such as ensuring privacy during interviews, securing storage of completed questionnaires, and preventing any sharing of data between outlets [32].

### Data analysis

Double data entry was conducted using Microsoft Access (Microsoft Corporation, Redmond, WA, USA) with built-in range and consistency checks. Data were analyzed across survey rounds using Stata Version 13.1 (StataCorp College Station, TX, USA).

Standard indicators were constructed according to definitions applied across the ACTwatch project described elsewhere [20, 33]. Anti-malarials identified during the outlet drug audit were classified according to information on drug formulation, active ingredients and strengths as non-artemisinin therapies, artemisinin monotherapies and ACT. ACT were classified as QAACT or non-QAACT, with the former including products meeting one of three criteria: (1) the product had WHO PQP status; (2) the product was in compliance with the Global Fund quality assurance policy and appears on the Global Fund list of approved products for procurement; or (3) the product was granted regulatory approval by the EMA. Products were matched to each of these lists across the categories of formulation, active ingredients, strength, manufacturer, country of manufacture, and package size. ACT that met all these conditions were classified as quality-assured ACT. Products that did not match all criteria were categorized as non-QAACT.

Availability was defined as the presence of one or more anti-malarials at the outlet at the time of the survey. The availability of specific anti-malarial categories was restricted to those outlets that had anti-malarials in stock. The availability of non-QAACT was measured as the proportion of outlets stocking non-QAACT, among all outlets with at least one anti-malarial in stock. Significant differences in non-QAACT availability levels between baseline year and most recent survey year in each country were estimated using logistic regression with survey settings, with a binary dependent variable for availability of non-QAACT at the outlet level, and a dummy independent variable for year. Types of non-QAACT found in the public and private sector were described using descriptive statistics for product information, including product generic name, formulation, country of manufacture, and national registration status.

The sales or distribution of the anti-malarials recorded in the drug audit were standardized using the adult equivalent treatment dose (AETD) to allow meaningful comparisons between anti-malarials with different treatment courses. The AETD is defined as the amount of active ingredient required to treat an adult weighing 60 kg according to WHO treatment guidelines. Median private sector price for one AETD was calculated for non-QAACT and for QAACT. The interquartile range (IQR) is displayed as a measure of dispersion. Price data presented were collected in local currencies and deflated to 2009 prices using national consumer price indices. Price data were converted to US dollars using official exchange rates for the data collection period obtained from http://www.oanda.com. Price measures included tablet anti-malarials only, given differences in unit costs for tablet and non-tablet formulations. While all QA ACT are by definition tablet formulation, non-QAACT are also available in non-tablet formulations, most commonly suspensions. The median price for one bottle for suspension formulation is also reported.

Provider reports on the amount of the drug sold or distributed during the week preceding the survey were used to calculate sales volumes according to type of anti-malarial. The volume of each drug is, therefore, the number of AETDs that were reportedly sold/distributed during the week preceding the survey. Measures of volume include all dosage forms to provide a complete assessment of anti-malarial market shares to the consumer or patient. Additional public health facilities and pharmacies sampled as part of over-sampling for these outlet types were not included in market share calculations. The statistical significance of differences in market share of non-QAACT was estimated using Stata’s ratio command, with survey settings, and the post-estimation ‘lincom’ (linear combination) command.

Sampling weights were calculated as the inverse of the probability of cluster selection. All point estimates were weighted using survey settings and all standard errors were calculated taking account of the clustered and stratified sampling strategy with Stata survey commands.

## Results

A total of 200,509 outlets were screened to assess availability of anti-malarials across the eight country contexts (Benin, Kinshasa and Katanga, Kenya, Madagascar, Nigeria, Tanzania, Uganda, and Zambia) and 29 survey rounds between 2009 and 2015 (Table 1). An audit of all available anti-malarial medicines was completed in 49,554 eligible outlets. In total, 336,017 anti-malarials were audited, including 78,558 QAACT and 83,130 non-QAACT. A catalogue of all the non-QAACT products audited can be found in Additional file 1.

**Table 1:** Results of the outlet census and anti-malarial audit by country and survey year

Country	Year	Screened (N outlets)	Anti-malarial audit completed (N outlets)	Anti-malarials audited (N drugs)	QAACT audited (N drugs)	Non-QAACT audited (N drugs)
West and Central Africa						
Benin	2009	1670	844	5233	859	1629
	2011	2891	1237	8987	1396	3925
	2014	4332	1806	14,378	2483	6454
DRC, Kinshasa	2009	2368	766	8437	151	1911
	2013	3364	931	12,291	216	6022
	2015	1168	1056	16,287	853	7389
DRC, Katanga	2013	2270	771	6493	854	1975
	2015	1052	993	8050	1507	2025
Nigeria	2009	5456	2113	20,841	1192	4624
	2011	7938	1486	13,391	2119	1610
	2013	5148	1714	14,358	4799	2058
	2015	13,483	3473	33,539	9586	7173
East Africa						
Kenya	2010	13,897	1888	8376	2052	2276
	2011	11,383	1854	9544	3669	2153
	2014	12,676	2133	9899	3234	3133
Tanzania	2010	3120	624	5544	416	1415
	2011	3702	787	9701	2045	2300
	2014	4724	2129	17,307	4905	2314
Uganda	2010	11,153	2410	14,427	2893	3785
	2011	16,207	3138	20,283	5495	4683
	2013	7932	3307	19,777	7182	4314
	2015	9438	4328	26,640	7380	7238
Southern Africa						
Madagascar	2010	6769	2414	5579	1790	184
	2011	10,046	2360	7234	3233	172
	2013	10,149	1756	6101	3851	116
	2015	13,481	1040	3170	1501	4
Zambia	2009	3378	435	1783	601	158
	2011	5436	781	3355	1036	594
	2014	5878	980	5012	1260	1496

### Availability of quality-assured and non-QAACT

**Fig. 3:**
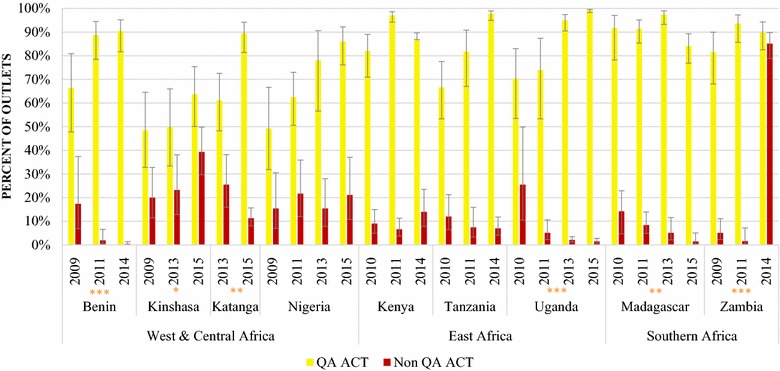
Availability of QAACT and non-QAACT in the public sector. Significant difference in non-QAACT availability between first and final survey year: *p < 0.05, **p < 0.01, ***p < 0.001

**Fig. 4:**
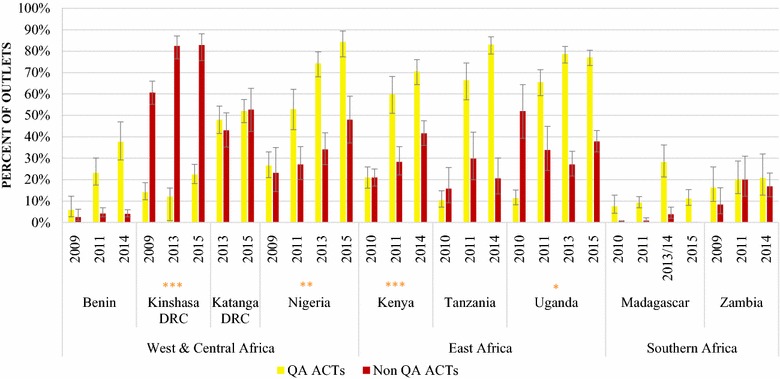
Availability of QAACT and non-QAACT in the private sector. Significant difference in non-QAACT availability between first and final survey year: *p < 0.05, **p < 0.01, ***p < 0.001

Figures 3 and 4 illustrate the availability of QAACT and non-QAACT in public and private sector anti-malarial stocking outlets. Non-QAACT availability in the public sector decreased significantly over time in Benin (2009, 17.4%; 2014, 0.5%; p < 0.001), Katanga (2013, 25.5%; 2015, 11.3%; p < 0.01), Uganda (2010, 25.5%; 2015, 1.5%; p < 0.001) and Madagascar (2010, 14.2%; 2015, 1.5%; p < 0.01) (Fig. 3). At the time of the most recent survey, availability was also relatively low in Tanzania (7.0%), Kenya (14.0%) and Nigeria (21.1%). Availability was high in Kinshasa (39.3%) in 2015, representing a significant increase from 20.0% in 2009 (p < 0.05). Availability was notably highest in Zambia at 85.1% in 2014, representing a significant increase from 5.1% in 2009 (p < 0.001).

Non-QAACT availability in the private sector increased significantly over time in Kinshasa (2009, 60.7%; 2015, 82.8%; p < 0.001), Nigeria (2009, 23.2%; 2015, 48.0%; p < 0.01), and Kenya (2010, 21.0%; 2015, 41.6%; p < 0.001) (Fig. 4). Availability decreased significantly in Uganda from 52.0% in 2010 to 37.8% in 2015 (p < 0.05). Private sector availability of non-QAACT varied substantially between countries during the most recent survey round from 0% in Madagascar and 4.0% in Benin, to 16.9% in Zambia, 20.6% in Tanzania, and 37.8% in Uganda. More than 40% of private sector outlets were stocking non-QAACT in Kenya (41.6%), and about half of outlets had non-QAACT in stock in Nigeria (48.0%) and Katanga (52.7%). Availability was 82.8% in Kinshasa.

Overall, QAACT availability was substantially higher than non-QAACT availability in the public and private sectors, particularly during the most recent survey round. Exceptions include Zambia, where availability of QAACT and non-QAACT was similar in 2014 in the public sector (QAACT, 89.8%; non-QAACT, 85.1%) and the private sector (QAACT, 20.8%; non-QAACT, 16.9%), and in Katanga where private sector availability was similar in 2015 (QAACT, 52.0%; non-QAACT, 52.7%). In Kinshasa, private sector non-QAACT availability has been substantially higher than QAACT availability across survey rounds. In 2015 QAACT availability was only 22.4% compared to 82.8% for non-QAACT.

### Characteristics of non-QAACT available in the public and private sector in the most recent survey round

Tables 2 and 3 describe public sector and private sector non-QAACT product information in studies with at least 25 non-QAACT audited within the sector. Across survey country contexts, nine different generic non-QAACT were audited in the public and private sector. The most common non-QAACT audited in both sectors in most countries was artemether-lumefantrine (AL). Dihydroartemisinin–piperaquine (DHA PPQ) was also common in certain countries, and in the public and private sectors in Kenya, Tanzania and Uganda, DHA PPQ was as common, or more common than AL. The majority of non-QAACT audited were tablet formulation across countries and sectors. However, suspensions were also common, accounting for approximately half or more of the audited products in the public sector in Kinshasa (68.7%) and Nigeria (45.8%), and about one-third of the products in the private sector in Kinshasa (39.8%), Katanga (33.9%), Nigeria (35.8%) and Kenya (30.4%).

**Table 2:** Characteristics of non-QAACT available in the public sector during the most recent survey round

	Kinshasa, DRC 2015	Katanga DRC 2015	Nigeria 2015	Kenya 2014	Tanzania 2014	Uganda 2015	Zambia 2014
	% (95% CI)	% (95% CI)	% (95% CI)	% (95% CI)	% (95% CI)	% (95% CI)	% (95% CI)
	N = 390	N = 148	N = 96	N = 50	N = 58	N = 59	N = 941
Active ingredients (type)							
Artemether lumefantrine	75.0 (69.9–79.5)	76.8 (66.1, 84.9)	76.2 (52.7, 90.2)	44.2 (23.4, 67.3)	52.6 (33.1, 71.4)	37.6 (22.1, 56.2)	100.0 (99.8, 100.0)
Artesunate amodiaquine	4.9 (3.1, 7.9)	10.5 (3.9, 25.3)	8.3 (1.6, 33.0)	0.0 (–)	0.0 (–)	0.0 (–)	0.0 (–)
Artesunate mefloquine	0.0 (–)	0.0 (–)	0.3 (<0.1, 2.2)	0.3 (0.1, 1.0)	5.0 (1.2, 18.0)	0.0 (–)	0.0 (–)
Artemisinin piperaquine	1.2 (0.3, 4.5)	1.6 (0.5, 4.9)	0.0 (–)	6.4 (2.8, 13.9)	2.5 (1.0, 6.3)	0.0 (–)	0.0
Artemisinin naphthoquine	0.9 (0.1, 6.1)	0.0 (–)	0.0 (–)	6.2 (2.6, 13.9)	0.0 (–)	19.0 (7.7, 39.7)	0.0 (–)
Artesunate SP	5.1 (3.3, 7.9)	5.5 (2.9, 10.3)	3.2 (0.5, 18.8)	0.8 (0.2, 3.0)	0.0 (–)	0.0 (–)	0.1 (<0.1, 0.2)
Dihydroartemisinin piperaquine	12.1 (8.2, 17.3)	2.1 (0.9, 4.7)	12.0 (4.4, 28.7)	42.1 (26.1, 59.9)	39.9 (22.0, 61.0)	43.0 (26.1, 61.6)	0.0 (–)
Dihydroartemisinin SP	0.8 (0.4, 1.8)	3.5 (0.9, 12.3)	0.0 (–)	0.0 (–)	0.0 (–)	0.0 (–)	0.0 (–)
Arterolane piperaquine	0.0 (–)	0.0 (–)	0.0 (–)	0.0 (–)	0.0 (–)	0.4 (0.1, 2.6)	0.0 (–)
Product formulation							
Tablet	56.5 (48.0, 64.7)	70.0 (61.4, 77.3)	61.0 (41.9, 77.2)	77.1 (64.5, 86.2)	91.5 (78.6, 96.9)	87.3 (74.7, 94.1)	99.6 (99.0, 99.8)
Suspension	43.5 (35.3, 52.0)	30.0 (22.7, 38.6)	38.1 (22.1, 57.2)	22.9 (13.8, 35.5)	7.6 (2.6, 20.1)	12.8 (5.9, 25.3)	0.4 (0.2, 1.0)
Granule	0.0 (–)	0.0 (–)	0.9 (0.1, 7.0)	0.0 (–)	0.9 (0.1, 5.6)	0.0 (–)	0.0 (–)

	N = 384	N = 145	N = 96	N = 50	N = 54	N = 59	N = 939
Country of manufacture^a^							
Local	25.6 (19.8, 32.4)	23.7 (15.9, 33.7)	17.1 (5.2, 43.5)	14.8 (7.1, 28.2)	1.1 (0.2, 6.6)	0.0 (–)	0.0 (–)
India	62.7 (57.2, 67.8)	69.7 (59.7, 78.1)	61.5 (42.8, 77.3)	58.8 (40.5, 75.0)	35.3 (18.2, 57.2)	37.7 (21.9, 56.5)	99.9 (99.7, 100.0)
China	2.4 (0.9, 6.6)	1.8 (0.6, 5.7)	19.0 (7.9, 39.0)	16.7 (8.7, 29.5)	47.3 (27.7, 67.7)	54.1 (33.9, 73.0)	0.0 (–)
European Country	3.4 (2.0, 5.6)	2.9 (1.3, 6.4)	0.3 (< 0.1, 2.2)	9.7 (5.5, 16.6)	16.3 (6.4, 35.6)	8.3 (2.8, 22.0)	0.1 (< 0.1, 0.4)
Other^b^	6.0 (2.7, 12.8)	1.9 (0.4, 9.3)	2.2 (0.4, 12.1)	0.0 (–)	0.0 (0)	0.0 (–)	0.0 (–)
Registered with the National Drug Regulatory Authority	52.8 (44.0, 61.4)	19.3 (13.2, 27.2)	48.5 (26.9, 70.6)	33.6 (21.5, 48.2)	n/a	63.2 (43.5, 79.2)	1.6 (0.9, 3.0)

Countries excluded include: Benin (n = 18 products) and Madagascar (n = 4 products)

*n/a* Registration list not available in Tanzania for 2014

^a^ Excluding products with unknown country of manufacture

^b^ Kinshasa: Vietnam; Katanga: Morocco; Nigeria: Morocco, Pakistan

**Table 3:** Characteristics of non-QAACT available in the private sector during the most recent survey round

	Benin 2014	Kinshasa, DRC 2015	Katanga, DRC 2015	Nigeria 2015	Kenya 2014	Tanzania 2014	Uganda 2015	Zambia 2014
	% (95% CI)	% (95% CI)	% (95% CI)	% (95% CI)	% (95% CI)	% (95% CI)	% (95% CI)	% (95% CI)
	N = 6436	N = 6999	N = 1877	N = 7077	N = 3083	N = 2255	N = 7179	N = 555
Active ingredients (type)								
Artemether lumefantrine	64.9 (61.6, 68.1)	67.0 (64.8, 69.2)	75.1 (68.0, 81.1)	65.8 (62.4, 69.0)	40.3 (36.5, 44.2)	29.0 (24.7, 33.7)	52.5 (48.5, 56.4)	75.4 (66.4, 82.6)
Artesunate amodiaquine	1.4 (1.0, 2.1)	3.8 (3.1, 4.6)	3.7 (2.9, 4.9)	5.5 (3.7, 8.1)	0.3 (0.2, 0.5)	0.2 (0.1, 0.3)	0.3 (0.1, 1.0)	1.1 (0.2, 7.5)
Artesunate mefloquine	5.5 (4.3, 7.1)	0.5 (2.6, 0.8)	<0.1 (<0.1, 0.2)	1.4 (0.7, 2.8)	2.8 (2.2, 3.6)	8.2 (5.7, 11.5)	1.1 (0.8, 1.7)	0.0 (–)
Artemisinin piperaquine	0.2 (0.0, 0.8)	1.4 (0.9, 2.1)	1.5 (0.6, 3.5)	0.8 (0.4, 1.6)	8.5 (7.9, 9.3)	19.3 (16.5, 22.4)	0.0 (–)	0.0 (–)
Artemisinin naphthoquine	1.1 (0.7, 1.5)	0.0 (–)	0.0 (–)	0.0 (–)	2.3 (1.9, 2.7)	1.7 (0.6, 4.6)	6.7 (5.4, 8.3)	0.7 (0.3, 1.5)
Artesunate SP	8.6 (7.6, 9.7)	9.7 (8.6, 10.8)	7.3 (4.0, 13.0)	1.6 (1.1, 2.4)	3.3 (2.6, 4.1)	0.0 (–)	0.0 (–)	21.2 (14.1, 30.5)
Dihydroartemisinin piperaquine	11.7 (9.2, 14.6)	14.6 (13.4, 15.8)	5.1 (3.8, 6.8)	24.7 (22.7, 46.7)	42.2 (40.0, 44.9)	41.7 (37.9, 45.7)	39.1 (36.0, 42.2)	1.6 (14.1, 30.5)
Dihydroartemisinin piperaquine trimethoprim	3.4 (2.9, 3.9)	0.2 (0.1, 0.5)	0.0 (–)	0.1 (<0.1, 0.2)	0.0 (–)	0.0 (–)	0.0 (–)	0.0 (–)
Dihydroartemisinin SP	3.3 (2.6,4.3)	2.9 (2.5, 3.3)	7.2 (6.3, 8.2)	0.0 (–)	0.0 (–)	0.0 (–)	0.0 (–)	0.0 (–)
Arterolane piperaquine	0.0 (–)	0.0 (–)	0.0 (–)	0.2 (0.1, 0.4)	0.0 (–)	0.0 (–)	0.3 (0.1, 0.5)	0.0 (–)
Product formulation								
Tablet	71.2 (68.3, 74.0)	60.2 (57.5, 62.8)	66.1 (63.9, 68.1)	60.8 (57.3, 64.2)	68.5 (66.2, 70.7)	86.4 (82.9, 89.2)	74.7 (71.3, 77.9)	74.3 (67.5, 80.1)
Suspension	25.3 (23.8, 26.9)	39.8 (37.2, 42.5)	33.9 (31.8, 36.1)	35.8 (31.9, 39.9)	30.4 (28.1, 32.9)	13.6 (10.8, 17.0)	25.2 (22.0, 28.6)	25.7 (19.9, 32.5)
Granule or suppository	3.5 (1.5, 7.9)	<0.1 (<0.1, 0.2)	<0.1 (<0.1, 0.2)	3.4 (2.6, 4.5)	1.1 (0.8, 1.6)	0.1 (<0.1, 0.2)	0.1 (0.1, 0.3)	0.0 (–)

	N = 6426	N = 6986	N = 1853	N = 7074	N = 3072	N = 2237	N = 7137	N = 546
Country of manufacture^a^								
Local	0.0 (–)	24.8 (23.4, 26.2)	29.2 (26.9, 31.5)	15.2 (13.1, 17.6)	3.9 (3.3, 4.7)	9.2 (6.7, 12.6)	1.3 (0.9, 1.9)	0.0 (–)
India	55.1 (53.1, 57.0)	60.5 (58.2, 62.8)	59.1 (51.7, 66.2)	59.3 (54.1, 64.4)	53.0 (51.3, 54.7)	6.5 (4.7, 8.9)	72.4 (69.3, 75.2)	67.9 (53.1, 79.8)
China	18.4 (17.3, 19.6)	2.5 (1.8, 3.4)	2.6 (1.5, 4.3)	21.3 (18.0, 25.1)	27.0 (25.2, 28.8)	62.0 (56.9, 66.8)	23.1 (20.5, 25.9)	4.3 (1.8, 9.7)
European Country	19.5 (17.8, 21.3)	7.6 (6.7, 8.7)	8.3 (3.7, 17.5)	2.2 (1.0, 4.8)	14.3 (13.0, 15.7)	22.1 (18.5, 26.3)	3.2 (2.3, 4.3)	27.8 (18.3, 39.8)
Other^b^	7.1 (5.7, 8.9)	4.6 (4.1, 5.1)	0.8 (0.3, 2.5)	2.0 (1.0, 4.1)	1.8 (1.3, 2.4)	0.2 (0.1, 0.7)	0.1 (<0.1, 0.4)	<0.1 (<0.1, 0.3)
Registered with a National Drug Regulatory Authority	36.4 (32.8, 40.3)	53.1 (52.0, 54.1)	41.6 (33.5, 50.3)	60.5 (57.6, 63.4)	64.7 (63.4, 66.1)	n/a	89.6 (87.8, 91.2)	79.5 (70.9, 86.1)

Countries excluded include: Madagascar (n = 0 products)

*n/a* Registration list not available in Tanzania for 2014

^a^ Excluding products with unknown country of manufacture

^b^ Benin: Cote d’Ivoire, DRC, Morocco, Nigeria, Senegal, Togo; Kinshasa: Vietnam; Katanga: Zambia; Nigeria: Malaysia, Morocco, Pakistan, Senegal, Vietnam; Kenya: Bangladesh, USA, Vietnam; Tanzania: Morocco, USA; Uganda: Morocco, USA; Zambia: Kenya

Non-QAACT were usually imported from other countries, although local manufacturing accounted for approximately one-quarter of audited products in the public and private sectors in Kinshasa (25.6% and 24.8%, respectively) and Katanga (23.7, 29.2% respectively). Products imported from India accounted for the majority of audited non-QAACT in both the public and private sectors in most countries, with the exception of a high proportion of products imported from China in Tanzania’s public and private sectors, and in Uganda’s public sector.

In total, over 180 unique manufacturers were identified. The number of unique manufacturers with more than one non-QAACT product audited in each country setting was as follows: Benin, 42; DRC, 45; Nigeria, 92; Kenya, 24; Tanzania, 19; Uganda, 9; Zambia, 16. In contrast, the number of unique manufacturers for quality-assured ACT audited in each country was considerably lower: Benin, 7; DRC, 6; Nigeria, 7; Kenya, 7; Tanzania, 6; Uganda, 7; Zambia 5.

The extent to which audited non-QAACT were registered by the national drug regulatory authority (NDRA) varied by country and sector. Half or more of audited non-QAACT in the most recent survey were registered with a NDRA in the public and private sectors of Nigeria (48.5, 60.5% respectively) and Uganda (63.2, 89.6% respectively), in the public sector in Kinshasa (52.8%), and in the private sector in Katanga (53.1%), Kenya (64.7%) and Zambia (79.5%). Notably, the ACT procured and made widely available in the public sector in Zambia (AL manufactured by S Kant.) was not found on the NDRA registration list.

### Price of QAACT and non-QAACT

**Fig. 5:**
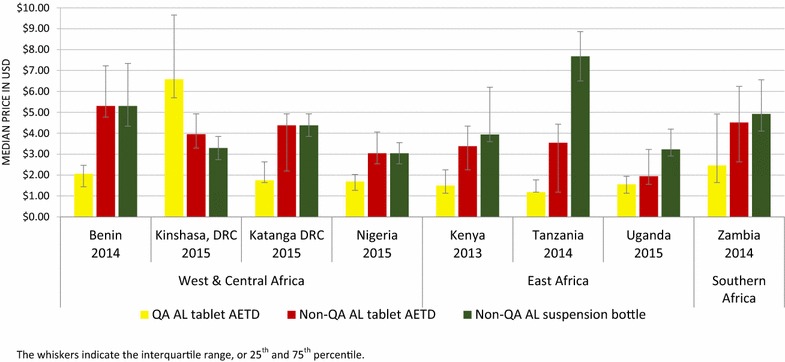
Private sector median price of QAACT and non-QAACT AL. The whiskers indicate the interquartile range, or 25th and 75th percentile

Figure 5 summarizes the median private sector price for one AETD for tablet formulation of quality-assured and non quality-assured AL, and suspension formulation of non quality-assured AL. Price is reported for AL, given that AL is a national first-line treatment and was the most common ACT audited within each country.

The median private sector price for non quality-assured AL tablets was from 1.3 (Uganda) to 3 (Tanzania) times higher than the price of quality-assured AL in all countries with the exception of Kinshasa, where quality-assured AL was 1.7 times more expensive than non quality-assured AL. Similarly, the price of one bottle of non quality-assured AL AETD suspension was between 1.8 (Nigeria) to 6.5 (Tanzania) times more expensive than one quality-assured AL AETD in all countries except Kinshasa where one quality-assured AL AETD was 2 times more expensive than one bottle of non quality-assured AL suspension.

The differences in price of quality-assured and non-QAACT for generics other than AL followed a similar pattern, whereby non-QAACT tablets and suspensions were more expensive than quality-assured tablets in each country (Additional file 2).

### Non-QAACT market share

**Fig. 6:**
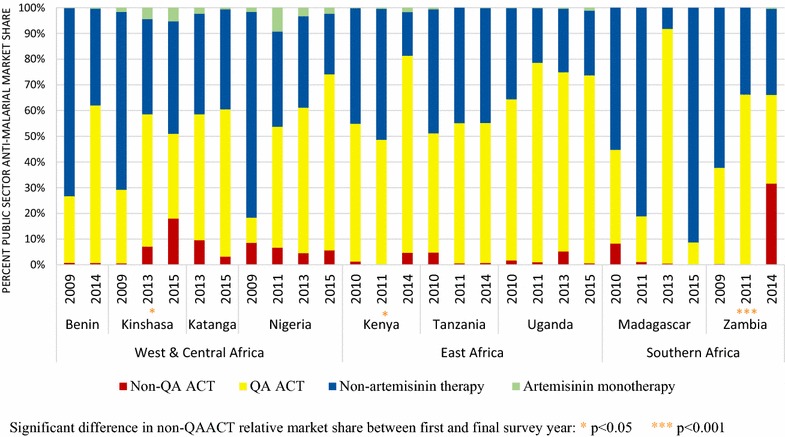
Anti-malarial market share within the public sector. Significant difference in non-QAACT relative market share between first and final survey year: *p < 0.05, ***p < 0.001

Among anti-malarials dispensed in the public sector, market share for non-QAACT increased significantly between the first and final survey years in Kinshasa (0.6–18.0%, p < 0.001), Kenya (1.3–4.7%, p < 0.01) and Zambia (0.4–31.6%, p < 0.001) (Fig. 6). Despite these significant increases, non-QAACT market share was low relative to the market share for QAACT and non-artemisinin therapies within the public sector in these countries, with the exception of Zambia where non-QAACT accounted for 31.6% of anti-malarials distributed in 2014. Aside from Zambia, non-QAACT market share in the public sector was highest in Kinshasa at 18.0% in 2015, and was non-negligible in Nigeria at 5.6% in 2015. Elsewhere, non-QAACT market share remained very low over time and was less than 1% during the most recent survey in Benin (0.7%), Tanzania (0.7%), Uganda (0.6%) and Madagascar (0.5%).

**Fig. 7:**
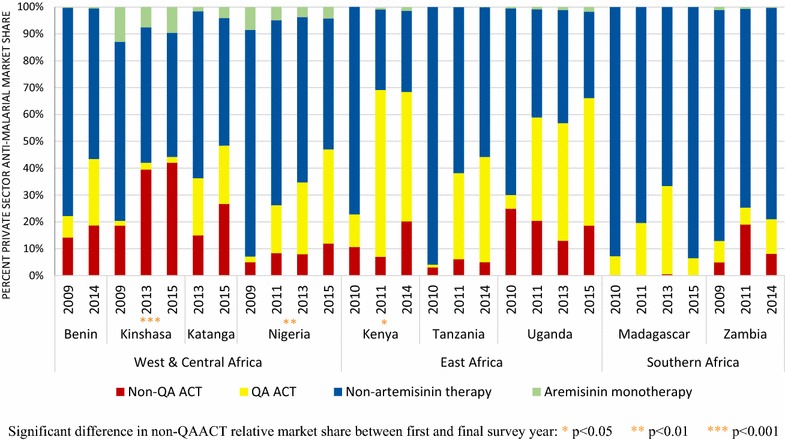
Anti-malarial market share within the private sector. Significant difference in non-QAACT relative market share between first and final survey year: *p < 0.05, **p < 0.01, ***p < 0.001

Market share for non-QAACT within the private sector increased significantly between first and final survey rounds in Kinshasa (18.6–42.0%, p < 0.001), Nigeria (5.0–12.0%, p < 0.01) and Kenya (10.7–20.2%, p < 0.05) (Fig. 7). At the time of the most recent survey round, non-QAACT market share was highest in Kinshasa, where 42.0% of all anti-malarials distributed by the private sector were non-QAACT, followed by Katanga (26.7% non-QAACT market share). Approximately one in five anti-malarials distributed by the private sector were non-QAACT in Benin (18.7%), Kenya (20.2%) and Uganda (18.6%). Approximately one in ten anti-malarials distributed by the private sector were non-QAACT in Nigeria (12.0%) and Zambia (8.1%). Non-QAACT market share was much lower in the private sectors of Tanzania (5.0%) and Madagascar (0.0%).

For ease of comparing the public and private sector findings, Additional file 3 illustrates a snapshot of the anti-malarial market share of non-QAACT between these sectors. The map illustrates how non-QAACT market share was higher in the private sector across all country contexts, with the exception of Zambia where 32% of the non-QAACT market share was through the public sector, compared to 8% in the private sector.

Non-QAACT market share differed across type of private sector outlet and tended to be highest among pharmacies as compared to other private sector outlet types. In the most recent survey round, non-QAACT market share within pharmacies ranged from one-quarter to one-third of all anti-malarial distribution in Kenya (24.6%), Tanzania (28.6%), Uganda (30.3%), Nigeria (39.7%), and Zambia (34.9%). Half or more of anti-malarials distributed by pharmacies were non-QAACT in Katanga (46.6%) and Benin (64.7%) (Additional file 4).

Tablets were the most commonly distributed non-QAACT formulation and accounted for greater than 75% of the private sector non-QAACT market share during the most recent survey round in each country (Benin, 89.7%; Kinshasa, 83.0%; Katanga, 88.1%; Kenya, 88.1%; Nigeria, 79.9%; Tanzania, 89.7%; Uganda, 93.6%; Zambia, 81.8%).

### Urban/rural location and private sector outlet type market share for non-QAACT

**Fig. 8:**
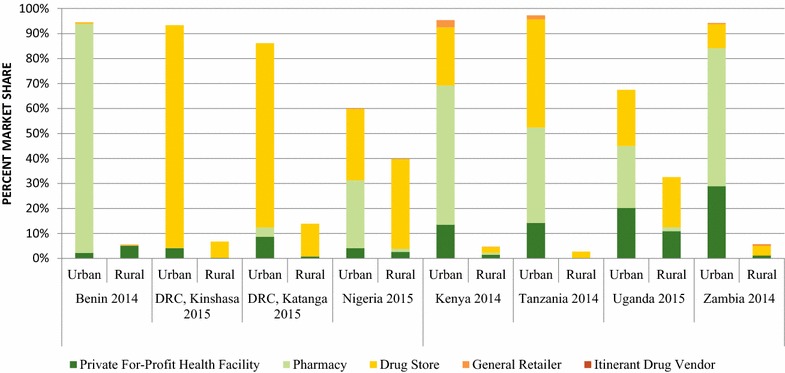
Urban rural and private sector outlet type market share for non-QAACT. Madagascar was not included as there were no quality-assured ACTs in the private sector in 2015 in Madagascar

Figure 8 shows the non-QAACT market share for each private sector outlet type and for urban and rural locations for the most recent survey rounds. Across studies, the majority of private sector non-QAACT were distributed in urban areas. More than 90% of non-QAACT were distributed through urban areas in Benin (94.5%), Kinshasa (93.3%), Kenya (95.4%), Tanzania (97.2%) and Zambia (94.2%). Urban distribution accounted for 86.1% of the market in Katanga, 67.4% in Uganda and 60.1% in Nigeria. Non-QAACT were distributed primarily by pharmacies or drug stores.

Additional file 5 shows the relative market share for all anti-malarials according to each private sector outlet type and for urban and rural locations for the most recent survey rounds. While urban outlets accounted for half or more of all anti-malarial distribution in each context with the exception of Nigeria (40.8%), urban market share for all anti-malarial distribution was lower than urban market share for non-QAACT across all contexts.

## Discussion

Non-QAACT form a substantial part of the anti-malarial market in sub-Saharan Africa. Of particular concern are Nigeria and the DRC, the countries with the highest malaria burden in the world [8] and where the private sector is responsible for the vast majority of anti-malarial distribution [34, 35]. In these two countries, half or more of all private sector anti-malarial stocking outlets had non-QAACT in stock, and non-QAACT accounted for one in ten anti-malarials distributed in Nigeria, one in four in Katanga DRC, and 40% of all anti-malarial distribution in Kinshasa DRC. Furthermore, non-QAACT availability and distribution have increased significantly in these countries in recent years. High private sector availability and distribution were noted in other malaria endemic countries, including Kenya and Uganda. Private sector availability was approximately 40% and non-QAACT accounted for one in five anti-malarials distributed in these countries. Results from this study provide key insights into non-QAACT markets with implications for policy and strategy.

### What do we know about non-QAACT on the market in sub-Saharan Africa?

#### Availability and variety

Overall, non-QAACT were commonly available in the private sector and were infrequently available and distributed within the public sector. Public sector availability was typically lower than 10% with notable exceptions in the DRC (Kinshasa, 39%) and Zambia (85%). Low public sector availability is likely a result of ACT procurements supported with donor funding and therefore subject to global quality-assurance standards. In Zambia, public sector procurement of non-QAACT from 2013 to 2014 was supported in part by government funding without the restrictions placed on donor-funded commodities [36]. Private sector availability of non-QAACT was generally higher than public sector availability with the exception of Zambia. While public sector outlets may be required to obtain particular drugs that meet certain quality standards, quality may not necessarily be a factor in private sector procurement decisions. The choice to stock a particular anti-malarial is likely to be influenced by competition spurred by the stocking trends of neighboring outlets, price, consumer demand, or consumer product perceptions [37].

The AMFm, first piloted and administered by the Global Fund, was designed to increase access to affordable QAACT for private sector first-line buyers. The approach increased the availability and market share for QAACT in the private sector in countries including four of the five AMFm countries studied here: Nigeria, Kenya, Tanzania and Uganda [27]. Results from this study found higher private sector availability of QAACT as compared with non-QAACT in these four countries, and in Madagascar (also an AMFm country) and Benin (a non-AMFm country with documented private sector availability of co-paid ACT due to leakage [38]). This was not the case in Zambia and the DRC (also a non-AMFm country), where non-QAACT  availability was higher than QAACT in the private sector. One might expect AMFm countries to have relatively low non-QAACT availability, but this was not always the case. Despite improvements in private sector QAACT availability in recent years, non-QAACT availability persists and furthermore, has increased in two of the AMFm countries with notable improvements in QAACT availability: Nigeria and Kenya.

Within the public and private sectors, nine different non-QAACT were identified across study countries, with AL being by far the most common, followed by DHA PPQ. AL is the most common first-line ACT in each study country, and was one of the first ACT medicines to be developed. A proliferation of non-QA AL products, including tablets and suspensions, is therefore not surprising. Other non-QAACT are relatively new combination therapies and have few, if any manufacturers with WHO prequalification or other stringent regulatory authority approval. This includes DHA PPQ, which in 2015 was manufactured by only one company with approval from WHO/Global Fund/EMA (Sigma Tau Pharmaceuticals Inc). Numerous brands of non-QAACT were identified, coming from over 180 distinct international manufacturers. Non-QAACT were most commonly available in tablet formulation, although suspensions were not uncommon, particularly in the DRC, Nigeria and Kenya where they accounted for one-third or more of audited non-QAACT products. Suspensions are designed and marketed for small children, given the challenge of administering tablets to infants and children.

#### Product location

Non-QAACT were usually distributed in urban areas, and disproportionately distributed in urban areas relative to all anti-malarial distribution. Non-QAACT were typically sold by pharmacies or drug stores, depending on the country context, and these outlet types were most commonly located in urban areas. Large urban pharmacies and drug stores are likely to have fewer barriers in procuring anti-malarials [39], including fewer supply chain levels to navigate [40], allowing for better access to a variety of products. In addition, outlets in urban environments typically serve wealthier customers who may be able to better afford the relatively high price of non-QAACT.

#### Price

One might have expected high relative distribution of non-QAACT to be driven by price considerations. However, results from this study show that non-QA tablets and suspensions were typically more expensive than QA AL tablets. The relatively low cost of QA first-line treatment is likely due to private sector subsidies implemented with support from the Global Fund. This copayment mechanism, first piloted as the AMFm, significantly reduced the cost of first-line QAACT in Kenya, Nigeria, Tanzania and Uganda [27]. This raises the question of why consumers would continue to pay more for non-QA products when cheaper QAACT are available. As noted earlier, products were primarily distributed by pharmacies and drug stores in urban areas, likely reflecting higher purchasing power of urban consumers. The phenomenon may be due in part to perceptions that higher prices are associated with higher quality, and/or consumer beliefs that subsidized ACT are of relatively poor quality [41], but additional research is needed to parse out purchasing determinants and consumer choice related to both price and tablet formulation. A better understanding of provider and consumer demand for QAACT and non-QAACT will be important for developing strategies to promote use of QA over non-QA products.

### Implications for anti-malarial drug policy and strategy

**Fig. 9:**
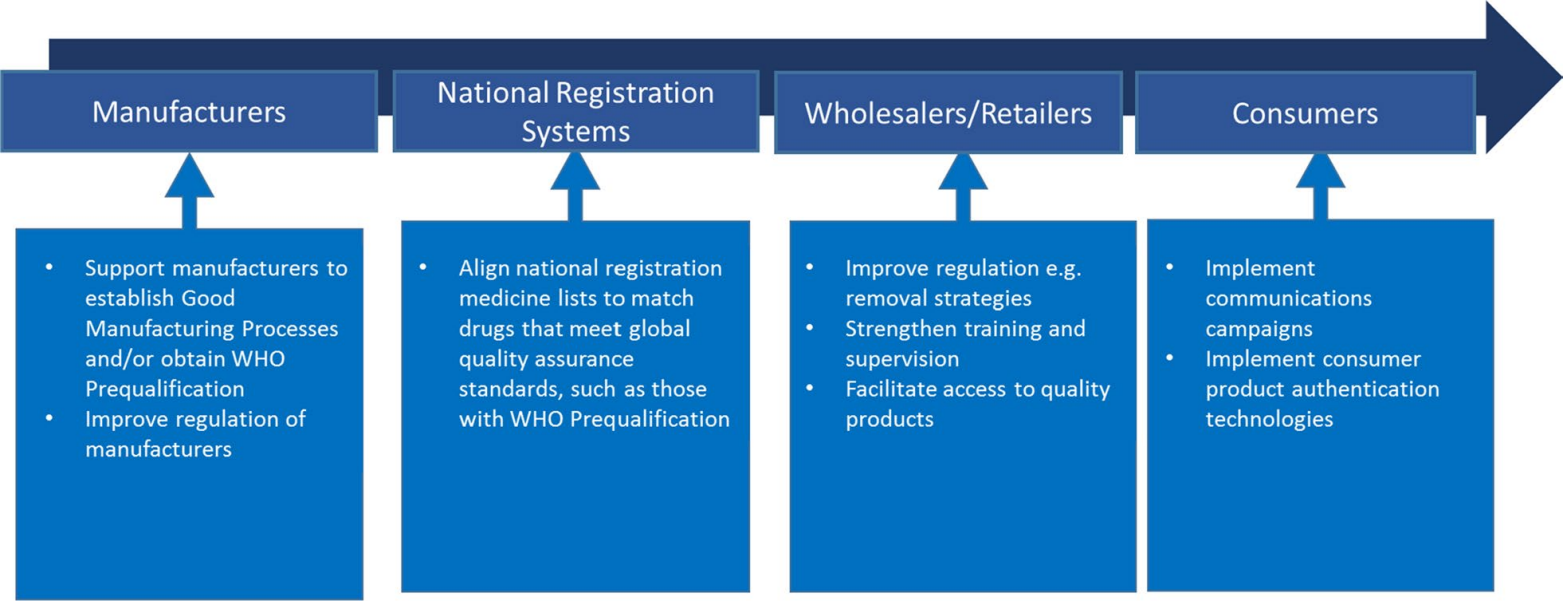
Opportunities for reducing non-QA product penetration in the supply chain

Addressing the availability and distribution of non-QAACT in sub-Saharan Africa will require strategies that target all levels of the anti-malarial supply and distribution chain. Figure 9 summarizes opportunities for reducing non-QAACT product penetration by targeting key elements of the supply chain: manufacturers, national registration systems, wholesalers and retailers, and consumers.

#### Manufacturers

This study defined QAACT according to global standards and prequalification/approval from the WHO, Global Fund, or EMA. During the most recent round of data collection in 2014/2015, only 12 manufacturers met QA standards and appeared on approved/prequalified anti-malarial drug lists for the WHO, the Global Fund, and/or the EMA. In contrast, 185 manufacturers of non-QAACT were identified across the eight study countries. For some of the products currently classified as non-QAACT, there may be potential for these products to achieve QA status by extending support to manufacturers to meet requirements for prequalification.

While the WHO PQP provides guidance and support for applicants, gaining approval from an external authority is a technically challenging, rigorous process that can take a minimum of three years. Fees for the application, in-country product registration, and facility GMP inspection can exceed $100,000 [42]. Combination therapies, such as those indicated for malaria, have particularly complex testing and regulatory requirements for approval. Even after achieving QA status, products are subject to periodic testing, monitoring, and re-approval. For small or newly established in-country manufacturers with limited resources, the barriers to this assessment and approval process are often prohibitive [23, 26]. Depending on the country, quality assurance activities may or may not be well-supported by the NDRA, the country-level drug licensing, control, and post-marketing surveillance body [21]. Supporting manufacturers to obtain GMP certification and apply for WHO PQP status could leverage existing resources to increase the presence of quality-assured drugs in malaria-endemic countries. Developing and enforcing national regulations for manufacturing quality would further ensure the production and supply of quality drugs. In the case of manufacturers who have not yet reached global quality standards, working to expand capacity for quality improvement may be most appropriate. Yet, maintaining high-quality and sustainable production in SSA presents its own challenges. The unstable energy supply, lack of technical specialists, and unpredictable transport systems characteristic of some SSA countries increase the likelihood of supply chain failures and can cause production costs to balloon [43, 44]. These challenges should be taken into consideration when designing and supporting viable, high-quality manufacturing sites.

It is also important to note that non-QAACT available in non-tablet formulations are unlikely to be eligible to obtain QA status. Suspensions involve reconstitution or volume measurement and this may inhibit accurate dosing. Additionally, once opened and reconstituted, the stability and hygiene of suspension formulations can no longer be guaranteed. As such, ACT suspensions are not included on the WHO pre-qualification or Global Fund procurement lists. A preferred pediatric-friendly alternative is the use of dispersible tablets which have been available for QA AL since 2009  [45, 46, 47]. There is need for additional information on consumer and provider preferences for ACT suspensions in the context of availability of dispersible tablets. Results from this study suggest that preference for suspensions may be driving availability in both private and public sectors in some countries.

#### National registration systems

Promoting the use of QAACT and discouraging the use of non-QAACT can be addressed at the national level, and can be facilitated through exclusion of non quality-assured products from national registration lists and government procurements. NDRA lists can be used to regulate private sector outlets by promoting products on the list and implementing communication, regulation and penalties regarding the importation and sale of non-registered products. However, NDRA lists in most of the study countries are currently not well-suited to this purpose as they are generally not aligned with global quality recommendations and national treatment guidelines. Results from this study found that typically, more than half of non-QAACT available were found on national-registration lists. Aligning national drug registration with global quality assurance standards may prove challenging given the need for multi-sectoral collaboration towards this effort. Where NDRA registration lists cannot be aligned with national or global standards for quality, national malaria control programs may need to devise independent approved lists of anti-malarials for training, supervision, communication and promotion purposes.

Active efforts to remove non-QAACT from anti-malarial markets could prove challenging. Efforts may not be readily acceptable to regulatory authorities and public and private buyers that have existing agreements in place with certain manufacturers or importers. Efforts to stop the importation and distribution of non-QAACT would also have economic consequences for manufacturers themselves. Local manufacturers, which were not uncommon in the DRC and Nigeria, may be particularly vulnerable to these potential economic impacts. Furthermore, a focus on removing non-QAACT must not preclude attention to removal of banned oral artemisinin monotherapies and ineffective non-artemisinin therapies that persist on the market to varying degrees in each of the countries included in this study [48]. Removal of these products may be of more imminent concern in certain areas than removal of non-QAACT, and thus, may need to be prioritized for regulatory attention. Public health policy and regulation shifts with respect to non-QAACT would require strategies that take all of these political and economic realities into account.

#### Wholesalers and retailers

Results from this study suggest the potential to substantially improve anti-malarial quality through greater private sector engagement and regulation to align private sector practices with national guidelines and quality-assurance standards. Private sector engagement to increase access to quality products can be facilitated in a variety of ways at the wholesaler and retailer level. Wholesalers, ranging from international importers to local merchandisers, can influence drug quality by restricting purchases to GMP-certified manufacturers and by monitoring products for non quality-assured drug removal. Improving private provider practices will also be an essential part of efforts to improve drug quality in the market. Strategies to improve provider practices in the private sector have included training, supervision and regulation under accreditation or other quality assurance programs. These programs are typically designed and implemented to promote the use of appropriate assessment, diagnostic/testing, referral, and treatment behaviors, including use of the first-line treatment for uncomplicated malaria, but they also provide an important opportunity for education and enforcement around non-QAACT. For example, a multi-pronged market intervention in Cambodia facilitated access to quality products through a diagnosis and treatment training for providers and through medical detailing that promoted quality assurance [49]. Tanzania’s Accredited Drug Dispensing Outlets (ADDO) pilot programme, which aimed to develop the capacity of pharmacy staff to provide quality drugs, was associated with a 13-fold reduction in unregistered medicines (from 26% at baseline to 2% after ADDO implementation) [50, 51]. Other private sector engagement initiatives, such as Nigeria’s patent medicines vendor training programme [52] and Kenya’s drug shop and clinic franchising programme [53, 54, 55, 56], have also led to quality improvement.

Results from this study show that strategies for private sector engagement and improved regulation can be highly targeted. The problem of non-QAACT availability and distribution is primarily in urban areas and is concentrated in many countries among either pharmacies or drug stores, though in other settings, private for-profit health facilities are also key outlets. Private sector engagement and regulatory enforcement focused on large urban outlets could be highly effective in removing these medicines from the market at national level. Private sector cooperation with law enforcement will be key in supporting these efforts. Conducting periodic site inspections to remove poor-quality products from the market and levying penalties for those who enable poor quality medicines to enter the market will help to improve overall market quality.

It should be noted that measures to remove non-QAACT from anti-malarial markets have the potential to reduce overall access to ACT. In the countries studied here, this is a risk primarily in the DRC, where availability of QAACT remains very low. In other study countries, particularly those with improved access to QAACT through public and private sector subsidy mechanisms, there would appear to be little to no risk in removing non quality-assured products from the shelves.

#### Consumers

The potential to stimulate demand for QAACT at the expense of non quality-assured medicines hinges on the ability of providers and consumers to identify and demand QA medicines. One approach to branding QAACT so that communications campaigns can promote their use is to use an identifying logo or quality seal. A green leaf logo placed on product packaging and promoted in mass media campaigns was used to identify co-paid QAACT under the AMFm and the subsequent Private Sector Copayment Mechanism. Awareness of the leaf, as evidenced by increases in uptake and availability of ACT, was well documented in countries that implemented mass media campaigns to promote the brand [57–59]. At the national level, a logo indicating quality and national approval could be applied to all QAACT and this logo could be used in campaigns to promote consumer trust and demand for these products.

An alternative to a quality logo is the use of mobile authentication systems (MAS). With MAS, scratch codes embedded on product packaging allow consumers to authenticate the product at the point of purchase via text message. Countries like Nigeria legally require all anti-malarials to carry a verifiable MAS code, which is only given to products registered with the national drug authority [60]. However, drug registration status may not be synonymous with drug quality and, until this is the case, MAS may provide a false sense of assurance for buyers. While mobile authentication has been useful for detecting falsified products and ensuring purchase of legitimate brands or nationally-registered products, it has yet to be used exclusively for product quality-assurance, such as tagging of WHO prequalified products.

#### Other strategies to improve anti-malarial quality

The above strategies are key for reducing the penetration of non-QAACT in the market. However, they are not sufficient on their own. They must be supported by additional, complementary measures to improve anti-malarial quality such as promoting GMP, improving drug testing capacity, encouraging proper drug transport and storage, and working with law enforcement to fight falsification. Efforts to increase the share of QAACT available and distributed to consumers are equally important, and engaging the private sector in these efforts will be paramount.

Many of the strategies discussed will rely on a strong NDRA. Strengthening regulatory capacity is also key for meeting external mandates for quality approval. NDRAs in SSA suffer from a host of common structural deficiencies, including a lack of technical guidelines, a chronic shortage of qualified medicine and facility assessors, limited legislative influence, regular use of adverse event-based response rather than risk-based quality monitoring systems, a general lack of accountability, and poor regulation enforcement [21]. Some countries have already taken steps towards implementing the strategies discussed. For example, Nigeria’s National Food and Drug Administration has undertaken drug screening through deployment of drug-authenticating Raman spectrometers [61]. In East Africa, six countries have banded together to improve drug regulation through the WHO/East African Community (EAC) Medicines Regulatory Harmonization Project. Other countries in Southeast Asia have coordinated with INTERPOL, customs authorities, and police to take action against poor quality medicines [4]. While efforts have been made in the public sector to ensure availability and distribution of QAACT, more effort is needed to address the issue in the private sector. Long-term quality improvement for anti-malarials will require a cross-sector, multiple-strategy approach.

### Study strengths and limitations

ACTwatch implements a rigorous, standardized methodology across study countries and over time. The outlet survey study design entails a full census of all outlets with the potential to distribute anti-malarials within selected clusters, and a full audit of all available products, thus constituting a study of the total anti-malarial market. The current findings are strengthened by measurement in multiple countries within west and central, east, and southern Africa with repeat cross-sections over time. Despite the strengths of the outlet survey design, certain limitations exist, including the potential that providers misreported stocking information or had poor recall, the cross-sectional nature of the surveys, and the possibility that in practice, certain outlets may have been missed despite the full census. These and other limitations are described extensively elsewhere [32, 33]. Specific to this study, surveys were powered to detect significant differences in availability of QAACT over time, whereas this study reported on non-QAACT indicators and therefore may not have had the power to detect meaningful change. In addition, chemical drug quality testing and analysis were beyond the scope of this project. Generalizability outside of the eight study countries is also likely to be limited, given that this study shows that non-QAACT product markets differ across countries, even within the same region.

Relying only on details of medicines recorded on audit sheets to define quality-assured status has limitations as any recording errors could lead to misclassification. Despite the intensive data collector training in this area, such errors do sometimes occur, for example, due to confusion over whether the country of manufacture or country of manufacturer headquarters should be recorded. Due to strict classification criteria, requiring all product fields to match those of global quality standard lists, a small proportion of products may have been incorrectly classified as a non-QAACT when they were in fact quality-assured.

## Conclusion

Non-QAACT are available and distributed to varying degrees in high malaria burden countries, primarily within the private sector and in urban areas. Non-QAACT availability and distribution were documented in settings with low private sector availability of QAACT including the DRC, as well as in countries with high private sector QAACT availability including Nigeria, Kenya and Uganda. The market is diverse, with multiple combinations from various manufacturers available in tablet and non-tablet formulations. Addressing the availability and distribution of non-QAACT will require effective private sector engagement and evidence-based strategies to address provider and consumer demand and supply. Given the variation in non-QAACT markets observed across eight countries, the design and implementation of efforts to limit registration, importation and distribution of non-QAACT must be tailored to the country context, and will no doubt involve addressing complex and challenging aspects of registration, private sector regulation, local manufacturing and drug importation. However, taking action to address non quality-assured medicine availability and use may be critical not only to patient health and safety, but to effective malaria control and protection of artemisinin and partner drug efficacy.

## Additional files

**Additional file 1.** Non-QAACT product catalogue.

**Additional file 2.** Median private sector price for QAACT and non-QAACT during the most recent survey round (US dollars).

**Additional file 3.** Snapshot of market share for non-QAACT across sectors in 8 study countries.

**Additional file 4.** Non-QAACT anti-malarial market share within each type of private sector outlet.

**Additional file 5.** Urban rural and private sector outlet type market share for all anti-malarials.

## Abbreviations

ACT: artemisinin-based combination therapy
ADDO: accredited drug dispensing outlet
AETD: adult equivalent treatment dose
AL: artemether-lumefantrine
AMFm: Affordable Medicines Facility-malaria
API: active pharmaceutical ingredient
DFID: Department for International Development
DHA-PPQ: dihydroartemisinin–piperaquine
DRC: Democratic Republic of the Congo
EAC: East African Community
EMA: European Medicines Agency
EOI: expression of interest
GMP: Good Manufacturing Practices
IQR: interquartile range
MAS: mobile authentication system
NDRA: National Drug Regulatory Authority
Non-QAACT: non quality-assured artemisinin combination therapy
PPS: probability proportional to size
QAACT: quality-assured artemisinin combination therapy
SSA: sub-Saharan Africa
WWARN: WorldWide Anti-malarial Resistance Network
WHO: World Health Organization
WHO PQP: World Health Organization’s Prequalification of Medicines Programme
