# Supplementary material for: Do anti-malarials in Africa meet quality standards? The market penetration of non quality-assured artemisinin combination therapy in eight African countries
Source: Malar J. 2017 May 25;16:204. doi: 10.1186/s12936-017-1818-8 (PMC5444102; doi:10.1186/s12936-017-1818-8)
Supplement: Supplementary file 1 — Additional file 1. Non-QAACT product catalogue. [file 12936_2017_1818_MOESM1_ESM.docx]

Additional File 1: Non-QAACT product catalogue

| **Strength** | **Manufacturer** | **Brand Name** | **Country of Manufacture** | **Benin** | **DRC** | **Kenya** | **Nigeria** | **Tanzania** | **Uganda** | **Zambia** |
| --- | --- | --- | --- | --- | --- | --- | --- | --- | --- | --- |
| **Artemether Lumefantrine Tablet** | | | | | | | | | | |
| 20/120 | Acton Pharma PVT LTD | Tonlum | India | x |  |  |  |  |  |  |
| 20/120 | Agog Pharma LTD | Co-Mether | India |  |  |  |  | x |  |  |
| 20/120 | Alisons SPRL | Co-Rimetar | India |  | x |  |  |  |  |  |
| 20/120 | Alkem Laboratories LTD | Chinther | India | x |  |  |  |  |  |  |
| 20/120 | Anhui Nhu Pharmaceuticals | Euromether | China |  |  |  | x |  |  |  |
| 20/120 | Astra Lifecare PVT LTD | Lumether | India |  | x |  |  | x |  | x |
| 20/120 | Aurochem Laboratories PVT LTD | Aurother | India |  |  |  |  | x |  |  |
| 20/120 | Aquila Biotech PVT LTD | Arlumetrin | India |  | x |  |  |  |  |  |
| 20/120 | Bharat Parenterals LTD | V-Martem | India | x |  |  | x |  |  |  |
| 20/120 | Bliss GVS Pharma LTD | Cofantrine | India | x | x |  |  |  |  |  |
| 20/120 | Bliss GVS Pharma LTD | Gvither Plus | India |  |  |  | x |  |  |  |
| 20/120 | Bliss GVS Pharma LTD | Lonart | India |  | x | x | x | x |  | x |
| 20/120 | Bliss GVS Pharma LTD | Lufanter | India | x |  |  |  |  |  |  |
| 20/120 | Bond Chemical IND LTD | Zoxine | Nigeria |  |  |  | x |  |  |  |
| 20/120 | Cachet Pharma PVT LTD | Cachart | India | x |  |  |  |  |  |  |
| 20/120 | Dawa LTD | Lum-artem | Kenya |  |  | x |  |  |  |  |
| 20/120 | Doctor Pharma Manufacturing UK LTD | Aludoc | India |  | x |  |  |  |  |  |
| 20/120 | Elys Chemical Industries LTD | Altem | Kenya |  |  | x |  |  |  |  |
| 20/120 | Emzor Pharma Industries LTD | Lokmal | Nigeria |  |  |  | x |  |  |  |
| 20/120 | Erica Healthcare PVT LTD | Lumether | India |  |  |  |  |  |  | x |
| 20/120 | Fiza Healthcare PVT LTD | Falciart | India | x |  |  |  |  |  |  |
| 20/120 | Glaxosmithkline Group | Colart | India | x | x |  | x |  |  |  |
| 20/120 | Globela Pharma PVT LTD | Lumaglobe | India |  |  |  | x |  |  |  |
| 20/120 | Ind-Swift LTD | Lamitar AM | India |  |  | x |  |  |  |  |
| 20/120 | Jenburkt Pharma LTD | Luma | India | x |  |  |  |  |  | x |
| 20/120 | Jiangsu Ruinian Qianjin Pharma Co LTD | Andermal | China |  |  |  | x |  |  |  |
| 20/120 | Jiangsu Ruinian Qianjin Pharma Co LTD | Sarmata | China |  |  |  | x |  |  |  |
| 20/120 | Jiangsu Ruinian Qianjin Pharma Co LTD | Sivometertrine | China |  |  |  | x |  |  |  |
| 20/120 | Jiangsu Ruinian Qianjin Pharma Co LTD | Coatal | China |  |  |  | x |  |  |  |
| 20/120 | LIC Pharma | Artrine | Ivory Coast | x |  |  |  |  |  |  |
| 20/120 | Lyn Edge Pharma LTD | Lynsunate | Nigeria |  |  |  | x |  |  |  |
| 20/120 | Magnolia LTD | Lumether | India |  |  |  |  |  |  | x |
| 20/120 | Mancare Pharma PVT LTD | Koyorine | India |  |  |  | x |  |  |  |
| 20/120 | Maxheal Laboratories PVT LTD | Famter | India |  |  |  | x |  |  |  |
| 20/120 | Maxheal Laboratories PVT LTD | Malanter | India |  |  |  | x |  |  |  |
| 20/120 | Maxheal Laboratories PVT LTD | Zeramal | India |  |  |  | x |  |  |  |
| **Strength** | **Manufacturer** | **Brand Name** | **Country of Manufacture** | **Benin** | **DRC** | **Kenya** | **Nigeria** | **Tanzania** | **Uganda** | **Zambia** |
| **Artemether Lumefantrine Tablet *(cont.)*** | | | | | | | | | | |
| 20/120 | MCW Healthcare LTD | Malaxit | India |  |  |  | x |  |  |  |
| 20/120 | Medinomics Healthcare PVT LTD | Fantem | India |  |  |  |  | x |  |  |
| 20/120 | Medreich LTD | Artrin | India | x |  |  | x | x |  |  |
| 20/120 | Micro Labs LTD | Amatem | India |  |  |  | x |  |  |  |
| 20/120 | Philco Pharma | Philco-Artelu | Germany | x |  |  |  |  |  |  |
| 20/120 | Rene Industries LTD | Lumaren | Uganda |  |  |  |  | x |  |  |
| 20/120 | S. Kant healthcare LTD | Artemether lumefantrine | India |  | x |  |  |  |  | x |
| 20/120 | S. Kant healthcare LTD | Cartef | India |  |  |  | x |  |  |  |
| 20/120 | Saga Laboratories | Pomex | India | x |  |  |  |  |  |  |
| 20/120 | Saga Laboratories | Romartef | India |  |  |  | x |  |  |  |
| 20/120 | Shalina Laboratories PVT LTD | L-Artem | India |  | x |  |  |  |  |  |
| 20/120 | Sharon Bio-Medicine LTD | Sharlum | India | x |  |  |  |  |  |  |
| 20/120 | Shelys Pharma LTD | Co-Malather | Tanzania |  |  |  |  |  | x |  |
| 20/120 | Sprukfield | Artel | Togo | x |  |  |  |  |  |  |
| 20/120 | Square Pharma LTD | Luteriam | Bangladesh |  |  | x |  |  |  |  |
| 20/120 | Stallion Laboratories PVT LTD | Havax | India |  |  |  | x |  |  |  |
| 20/120 | Swiss Pharma LTD | Arenax Plus | India |  |  |  | x |  |  |  |
| 20/120 | Swiss Pharma LTD | Nancimal | India |  |  |  | x |  |  |  |
| 20/120 | Tongmei Laboratories | AL Tong Mei | Togo | x |  |  |  |  |  |  |
| 20/120 | Universal Corporation LTD | Co-Max | Kenya |  |  | x |  |  |  | x |
| 20/120 | Yanzhou Xier Kangtai Pharma Co. LTD | Malastop | China |  |  |  | x |  |  |  |
| 20/120 | Yanzhou Xier Kangtai Pharma Co. LTD | Norinate DS | China |  |  |  | x |  |  |  |
| 20/120 | Zenufa Laboratories | Co-Artluf | India |  | x |  |  |  |  |  |
| 20/120 | Zest Pharma | Luther | India |  | x |  |  |  |  |  |
| 40/240 | Afrab-Chem LTD | La-Tesen | Nigeria |  |  |  | x |  |  |  |
| 40/240 | Ajanta Pharma LTD | Artefan | India | x |  | x | x |  |  | x |
| 40/240 | Alice Pharma PVT LTD | Artiz | India | x |  |  |  |  |  |  |
| 40/240 | Alkem Laboratories LTD | Chinther | India | x |  |  |  |  |  |  |
| 40/240 | Astamed Healthcare PVT LTD | Palumal | India |  |  |  | x |  |  |  |
| 40/240 | Bliss GVS Pharma LTD | Lonart | India |  | x | x |  | x |  | x |
| 40/240 | Bliss GVS Pharma LTD | Lufanter | India | x |  |  |  |  |  |  |
| 40/240 | Cipla LTD | Lumet Forte | India | x |  |  |  |  |  | x |
| 40/240 | Doctor Pharma Manufacturing UK LTD | Aludoc | India |  | x |  |  |  |  |  |
| 40/240 | Drugfield Pharma LTD | Drutemal Plus | Nigeria |  |  |  | x |  |  |  |
| 40/240 | Exus Pharmaceutical LTD | Exus Artemether | Nigeria |  |  |  | x |  |  |  |
| 40/240 | Generic Healthcare PVT LTD | Artrim | India | x |  |  |  |  |  |  |
| **Strength** | **Manufacturer** | **Brand Name** | **Country of Manufacture** | **Benin** | **DRC** | **Kenya** | **Nigeria** | **Tanzania** | **Uganda** | **Zambia** |
| **Artemether Lumefantrine Tablet *(cont.)*** | | | | | | | | | | |
| 40/240 | Global Pharma Healthcare PVT LTD | La-Forte | India |  | x |  |  |  |  |  |
| 40/240 | Gujarat Liqui Pharmacpas PVT LTD | Lumesoft Plus | India |  |  | x |  |  |  |  |
| 40/240 | Gujarat Liqui Pharmacpas PVT LTD | Uplufa Plus | India |  |  |  | x |  |  |  |
| 40/240 | Hovid BHD | Luter | Malaysia |  |  |  | x |  |  |  |
| 40/240 | Ipca Laboratories LTD | Laritem | India | x |  |  |  |  |  |  |
| 40/240 | Jiangsu Ruinian Qianjin Pharma Co LTD | Andermal | China |  |  |  | x |  |  |  |
| 40/240 | Jiangsu Ruinian Qianjin Pharma Co LTD | Tamether | China |  |  |  | x |  |  |  |
| 40/240 | LIC Pharma | Artrine | Ivory Coast | x |  |  |  |  |  |  |
| 40/240 | May & Baker Nigeria PLC | Artelum | Nigeria |  |  |  | x |  |  |  |
| 40/240 | Medreich LTD | Artrin | India | x |  |  |  |  |  |  |
| 40/240 | Mekophar Chemical Pharma Joint Stock Co | Artemether Plus | Vietnam |  |  |  | x |  |  |  |
| 40/240 | MSR Laboratories | Lumate | France | x |  |  |  |  |  |  |
| 40/240 | Nigeria Army Small Scale Manufacturing Unit | Nartel | Nigeria |  |  |  | x |  |  |  |
| 40/240 | Phatkin | Arthefan | DRC |  | x |  |  |  |  |  |
| 40/240 | Safi Pharma | Palufin | India |  | x |  |  |  |  |  |
| 40/240 | Saga Laboratories | Pomex | India |  | x |  |  |  |  |  |
| 40/240 | Shalina Laboratories PVT LTD | L-Artem | India |  | x |  |  |  |  |  |
| 40/240 | Sharon Bio-Medicine LTD | Sharlum | India | x |  |  |  |  |  |  |
| 40/240 | Zest Pharma | Luther | India |  | x |  |  |  |  |  |
| 80/480 | AC Drug LTD | Artemetrin | Nigeria |  |  |  | x |  |  |  |
| 80/480 | Acton Pharma PVT LTD | Tonlum | India | x |  |  |  |  |  |  |
| 80/480 | Adams Pharma Co LTD | Sumether-Plus | China |  |  |  | x |  |  |  |
| 80/480 | Ajanta Pharma LTD | Artefan | India | x |  | x |  | x | x | x |
| 80/480 | Ajanta Pharma LTD | Combisunate | India |  |  |  | x |  |  |  |
| 80/480 | Alice Pharma PVT LTD | Artiz | India | x |  |  |  |  |  |  |
| 80/480 | Alisons SPRL | Co-Rimetar | India |  | x |  |  |  |  |  |
| 80/480 | Astramed Healthcare PVT LTD | Palumal | India |  |  |  | x |  |  |  |
| 80/480 | Astra Lifecare PVT LTD | Lumether | India |  | x |  |  |  |  |  |
| 80/480 | Aurochem Laboratories PVT LTD | Aurother | India |  |  |  | x |  |  |  |
| 80/480 | Ave Pharma SPRL | Lufamet | DRC |  | x |  |  |  |  |  |
| 80/480 | Axelia Paloma Pharmax PVT LTD | Co-Arther | India |  | x |  |  |  |  |  |
| 80/480 | Baroque Pharma PVT LTD | Ravimal | India |  |  |  | x |  |  |  |
| 80/480 | Bliss GVS Pharma LTD | Cofantrine | India | x | x | x |  |  |  |  |
| 80/480 | Bliss GVS Pharma LTD | Gvither Plus | India |  |  |  | x |  |  |  |
| 80/480 | Bliss GVS Pharma LTD | Lonart | India |  | x | x | x | x | x |  |
| **Strength** | **Manufacturer** | **Brand Name** | **Country of Manufacture** | **Benin** | **DRC** | **Kenya** | **Nigeria** | **Tanzania** | **Uganda** | **Zambia** |
| **Artemether Lumefantrine Tablet *(cont.)*** | | | | | | | | | | |
| 80/480 | Cachet Pharma PVT LTD | Cachart | India | x |  |  |  |  |  |  |
| 80/480 | Cipla LTD | Artemef | India |  |  |  | x |  |  |  |
| 80/480 | Ciron Drugs & Pharma PVT LTD | Ronymal | India |  |  |  | x |  |  |  |
| 80/480 | Coral Laboratories LTD | Co-Corither | India |  |  | x |  |  |  |  |
| 80/480 | D-Glopa Pharma LTD | Ferife DS | Nigeria |  |  | x |  |  |  |  |
| 80/480 | Dabak Nigeria LTD | Co-Dabamal | India |  |  | x |  |  |  |  |
| 80/480 | Doctor Pharma Manufacturing UK LTD | Aludoc | India |  | x |  |  |  |  |  |
| 80/480 | Emzor Pharma Industries LTD | Lokmal | Nigeria |  |  |  | x |  |  |  |
| 80/480 | Fatoz Pharma PVT LTD | Faverid | India |  |  |  | x |  |  |  |
| 80/480 | Fidson Healthcare PLC | Arthemed | Nigeria |  |  |  | x |  |  |  |
| 80/480 | First Swiss Pharma SA | Nomal | India |  | x |  |  |  |  |  |
| 80/480 | Fiza Healthcare PVT LTD | Falciart | India | x |  |  |  |  |  |  |
| 80/480 | Fredun Pharma LTD | Asmether | India |  |  |  | x |  |  |  |
| 80/480 | Getz Pharma PVT LTD | Artheget | Pakistan |  |  |  | x |  |  |  |
| 80/480 | Glenmark Pharma LTD | Artiglen Max | India |  |  |  |  |  |  | x |
| 80/480 | Global Pharma Healthcare PVT LTD | LA-DS | India |  | x |  |  |  |  |  |
| 80/480 | Globela Pharma PVT LTD | Aquamal | India |  |  |  | x |  |  |  |
| 80/480 | Greenfiled Pharma (Jiangsu) Co. LTD | Atmal | China |  |  |  | x |  |  |  |
| 80/480 | Hovid BHD | Luter | Malaysia |  |  |  | x |  |  |  |
| 80/480 | Impact Healthcare PVT LTD | R-Lume | India | x |  |  |  |  |  |  |
| 80/480 | Indus Pharma PVT LTD | Indomal | Pakistan |  |  |  | x |  |  |  |
| 80/480 | West Coast Pharm Works LTD | Zymal | India |  |  |  | x |  |  |  |
| 80/480 | Ipca Laboratories LTD | Laritem | India | x |  |  |  | x |  |  |
| 80/480 | Ipca Laboratories LTD | Lumerax | India |  |  |  | x |  | x |  |
| 80/480 | Jiangsu Pengyao Pharma Co. LTD | Marleysunate DS | China |  |  |  | x |  |  |  |
| 80/480 | Jiangsu Ruinian Qianjin Pharma Co LTD | Andermal | China |  |  |  | x |  |  |  |
| 80/480 | Jiangsu Ruinian Qianjin Pharma Co LTD | Artborah | China |  |  |  | x |  |  |  |
| 80/480 | Jiangsu Ruinian Qianjin Pharma Co LTD | Hanmal | China |  |  |  | x |  |  |  |
| 80/480 | Jiangsu Ruinian Qianjin Pharma Co LTD | Hatherley | China |  |  |  | x |  |  |  |
| 80/480 | Jiangsu Ruinian Qianjin Pharma Co LTD | Meflax | China |  |  |  | x |  |  |  |
| 80/480 | Jiangsu Ruinian Qianjin Pharma Co LTD | Micpon | China |  |  |  | x |  |  |  |
| 80/480 | Jiangsu Ruinian Qianjin Pharma Co LTD | Pemametre | China |  |  |  | x |  |  |  |
| 80/480 | Jiangsu Ruinian Qianjin Pharma Co LTD | Sarmata | China |  |  |  | x |  |  |  |
| 80/480 | Jiangsu Ruinian Qianjin Pharma Co LTD | Sivometertrine | China |  |  |  | x |  |  |  |
| 80/480 | Jiangsu Ruinian Qianjin Pharma Co LTD | SMT Forte | China |  |  |  | x |  |  |  |
| 80/480 | Jiangsu Ruinian Qianjin Pharma Co LTD | Tamether | China |  |  |  | x |  |  |  |
| **Strength** | **Manufacturer** | **Brand Name** | **Country of Manufacture** | **Benin** | **DRC** | **Kenya** | **Nigeria** | **Tanzania** | **Uganda** | **Zambia** |
| 80/480 | Jiangsu Ruinian Qianjin Pharma Co LTD | Telufan Forte | China |  |  |  | x |  |  |  |
| 80/480 | Jiangsu Yixing Forwards Pharma Factory | Coatal | China |  |  |  | x |  |  |  |
| 80/480 | Jiangxi Xierkagtai Pharma Co. LTD | Artemefan | China |  |  |  | x |  |  |  |
| 80/480 | Kadark Pharma LTD | Athelutrin Forte | Nigeria |  |  |  | x |  |  |  |
| 80/480 | Kayhelt Pharma LTD | Kart-LU | India |  |  |  | x |  |  |  |
| 80/480 | Kilitch Drugs LTD | Artluf-Forte | India | x |  |  |  |  |  |  |
| 80/480 | Kwality Pharma LTD | Lasunate DS | India |  |  |  | x |  |  |  |
| 80/480 | LIC Pharma | Artrine | Ivory Coast | x |  |  |  |  |  |  |
| 80/480 | Lyn Edge Pharma LTD | Lynsunate | Nigeria |  |  |  | x |  |  |  |
| 80/480 | Maan Pharma LTD | Tama-ACT | India |  |  |  | x |  |  |  |
| 80/480 | Maxheal Laboratories PVT LTD | Famter | India |  |  |  | x |  |  |  |
| 80/480 | Maxheal Laboratories PVT LTD | Malanter | India |  |  |  | x |  |  |  |
| 80/480 | Maxheal Laboratories PVT LTD | Metherine Forte | India |  |  |  | x |  |  |  |
| 80/480 | Maxheal Laboratories PVT LTD | Zeramal | India |  |  |  | x |  |  |  |
| 80/480 | MCW Healthcare LTD | Fytama | India |  |  |  | x |  |  |  |
| 80/480 | MCW Healthcare LTD | Malaxit | India |  |  |  | x |  |  |  |
| 80/480 | Medical Pharma | Bimalaril | China | x |  |  |  |  |  |  |
| 80/480 | Medico Remedies PVT LTD | Artemetin Beta Plus | India |  |  |  | x |  |  |  |
| 80/480 | Mekophar Chemical Pharma Joint Stock Co | Artemether Plus | Vietnam |  |  |  | x |  |  |  |
| 80/480 | Micro Labs LTD | Amatem | India |  |  |  | x |  |  |  |
| 80/480 | Naxpar Laboratories PVT LTD | Fynale | India |  |  |  | x |  |  |  |
| 80/480 | New Cesamex | Cether-L | DRC |  | x |  |  |  |  |  |
| 80/480 | Nicholson Healthcare | Dosunate | India |  |  |  | x |  |  |  |
| 80/480 | Olive Healthcare | Grutha Forte | India |  |  |  | x |  |  |  |
| 80/480 | Omak Pharma LTD | Lactomak | India |  |  |  | x |  |  |  |
| 80/480 | Osaka Pharma PVT LTD | Co-Fanart | India |  |  |  | x |  |  |  |
| 80/480 | Osaka Pharma PVT LTD | Gloatem Forte | India |  |  |  | x |  |  |  |
| 80/480 | Phamatex Industries LTD | Lumapil Forte | Nigeria |  |  |  | x |  |  |  |
| 80/480 | Phatkin | Arthefan | DRC |  | x |  |  |  |  |  |
| 80/480 | Rhydburg Pharma LTD | Sivoplus | India |  |  |  | x |  |  |  |
| 80/480 | Richygold International LTD | Art-Luf Forte | Nigeria |  |  |  | x |  |  |  |
| 80/480 | S. Kant Healthcare LTD | Cartef | India |  |  |  | x |  |  |  |
| 80/480 | Safi Pharma | Palufin | India |  | x |  |  |  |  |  |
| 80/480 | Saga Laboratories | Crotan Forte | India |  |  |  | x |  |  |  |
| 80/480 | Saga Laboratories | Romartef | India |  |  |  | x |  |  |  |
| 80/480 | Salud Care | TLC-Ether Forte | India |  | x |  |  |  |  |  |
| 80/480 | Saokim Pharma JSC | Lufart QS | Vietnam |  |  |  | x |  |  |  |
| **Strength** | **Manufacturer** | **Brand Name** | **Country of Manufacture** | **Benin** | **DRC** | **Kenya** | **Nigeria** | **Tanzania** | **Uganda** | **Zambia** |
| 80/480 | Shaimil Laboratories | Larot | India |  |  |  | x |  |  |  |
| 80/480 | Shalina Laboratories PVT LTD | L-Artem | India |  | x |  |  |  |  |  |
| 80/480 | Shalina Laboratories PVT LTD | Shal’Artem | India |  |  |  | x |  |  |  |
| 80/480 | Sharon Bio-Medicine LTD | Sharlum | India | x |  |  |  |  |  |  |
| 80/480 | Shelys Pharma LTD | Co-Malather | Tanzania |  |  |  |  |  | x |  |
| 80/480 | Sifa Pharma | Arte-Ped | India |  | x |  |  |  |  |  |
| 80/480 | SKG Pharma LTD | Lumal DS | Nigeria |  |  |  | x |  |  |  |
| 80/480 | Sold Accord Healthcare LTD | Salumether | India |  |  |  | x |  |  |  |
| 80/480 | Stallion Laboratories PVT LTD | Arteforth | India |  |  |  | x |  |  |  |
| 80/480 | Stallion Laboratories PVT LTD | BG Mal | India |  |  |  | x |  |  |  |
| 80/480 | Stallion Laboratories PVT LTD | Havax | India |  |  |  | x |  |  |  |
| 80/480 | Stallion Laboratories PVT LTD | Robamal | India |  |  |  | x |  |  |  |
| 80/480 | Strides Arcolab LTD | Combiart | India | x |  |  |  |  |  |  |
| 80/480 | Swiss Pharma LTD | Arcofan | India |  |  |  | x |  |  |  |
| 80/480 | Swiss Pharma LTD | Arenax Plus | India |  |  |  | x |  |  |  |
| 80/480 | Swiss Pharma LTD | Diconart | India |  |  |  | x |  |  |  |
| 80/480 | Swiss Pharma LTD | Loceet Forte | India |  |  |  | x |  |  |  |
| 80/480 | Swiss Pharma LTD | Menfancil | India |  |  |  | x |  |  |  |
| 80/480 | Swiss Pharma LTD | Nancimal | India |  |  |  | x |  |  |  |
| 80/480 | Swiss Pharma LTD | Voather Forte | India |  |  |  | x |  |  |  |
| 80/480 | Swiss Pharma LTD | Winart | India |  |  |  | x |  |  |  |
| 80/480 | The Madras Pharmaceuticals | Nimartem | India |  |  |  | x |  |  |  |
| 80/480 | Vapi Care Pharma PVT LTD | Ogamal | India |  |  |  | x |  |  |  |
| 80/480 | Yanzhou Xier Kangtai Pharma Co. LTD | Arterine Forte | China |  |  |  | x |  |  |  |
| 80/480 | Yanzhou Xier Kangtai Pharma Co. LTD | Malastop | China |  |  |  | x |  |  |  |
| 80/480 | Yanzhou Xier Kangtai Pharma Co. LTD | Norinate DS | China |  |  |  | x |  |  |  |
| 80/480 | Yes Pharma International LTD | Yesmal Forte | Pakistan |  |  |  | x |  |  |  |
| 80/480 | Zenufa Laboratories | Co-Artluf | India |  | x |  |  |  |  |  |
| 80/480 | Zest Pharma | Luther | India |  | x |  |  |  |  |  |

| **Strength** | **Manufacturer** | **Brand Name** | **Country of Manufacture** | **Benin** | **DRC** | **Kenya** | **Nigeria** | **Tanzania** | **Uganda** | **Zambia** |
| --- | --- | --- | --- | --- | --- | --- | --- | --- | --- | --- |
| **Artemether Lumefantrine Suspension** | | | | | | | | | | |
| 15/90 | Astamed Healthcare PVT LTD | Gimeter | India |  |  |  | x |  |  |  |
| 15/90 | Astamed Healthcare PVT LTD | Palumal | India |  |  |  | x |  |  |  |
| 15/90 | Axelia Paloma Pharmax India PVT LTD | Co-Arther | India |  | x |  |  |  |  |  |
| 15/90 | Centurion Laboratories | Artemether Lumefantrine | India |  |  |  |  | x |  |  |
| 15/90 | Dawa LTD | Lum-Artem | Kenya |  |  | x |  |  |  |  |
| 15/90 | Fredun Pharma LTD | Asmether | India |  |  |  | x |  |  |  |
| 15/90 | Generic Healthcare PVT LTD | Artrim | India | x |  |  |  |  |  |  |
| 15/90 | Getz Pharma PVT LTD | Artheget | Pakistan |  |  |  | x |  |  |  |
| 15/90 | Macleods | Lumiter | India | x |  |  |  |  |  |  |
| 15/90 | Magnolia LTD | Lumether | India |  |  | x |  |  |  | x |
| 15/90 | Medipharm Industries LTD | Co-Malartem | Uganda |  |  |  |  | x |  |  |
| 15/90 | Pharm-Inter SPRL | Arlu | China |  | x |  |  |  |  |  |
| 15/90 | Sold Accord Healthcare LTD | Salumether | India |  |  |  | x |  |  |  |
| 15/90 | Umedica Laboratories PVT LTD | Lufenart | India |  |  | x |  |  |  |  |
| 15/90 | Universal Corporation LTD | Co-Max | Kenya |  |  | x |  |  |  |  |
| 20/120 | Adams Pharma Co LTD | Sumether-Plus | China |  |  |  | x |  |  |  |
| 20/120 | Afrab-Chem LTD | La-Tesen | Nigeria |  |  |  | x |  |  |  |
| 20/120 | Alisons SPRL | Co-Rimetar | India |  | x |  |  |  |  |  |
| 20/120 | Ally Pharma Options PVT LTD | Famter | India |  |  |  | x |  |  |  |
| 20/120 | Bliss GVS Pharma LTD | Lonart | India |  |  |  | x |  |  | x |
| 20/120 | Jiangsu Ruinian Qianjin Pharma Co LTD | Tamether | China |  |  |  | x |  |  |  |
| 20/120 | Jiangsu Yixing Forward Pharma Factory | Coatal | China |  |  |  | x |  |  |  |
| 20/120 | Medical Pharma | Bimalaril | China | x |  |  |  |  |  |  |
| 20/120 | Osaka Pharma PVT LTD | Co-Fanart | India |  |  |  | x |  |  |  |
| 180/1080 | AC Drugs LTD | Artemetrin | Nigeria |  |  |  | x |  |  |  |
| 180/1080 | Adams Pharma Co LTD | Sumether-Plus | China |  |  |  | x |  |  |  |
| 180/1080 | Agog Pharma LTD | India | Co-Mether |  |  |  |  | x |  |  |
| 180/1080 | Ajanta Pharma LTD | Artefan | India | x |  | x |  |  |  | x |
| 180/1080 | Alice Pharma PVT LTD | Artiz | India | x |  |  |  |  |  |  |
| 180/1080 | Alisons SPRL | Co-Rimetar | India |  | x |  |  |  |  |  |
| 180/1080 | Aquila Biotech PVT LTD | Arlumetrin | India |  | x |  |  |  |  |  |
| 180/1080 | Ave Pharma SPRL | Lufamet | DRC |  | x |  |  |  |  |  |
| 180/1080 | Avro Pharma LTD | Avromal | Nigeria |  |  |  | x |  |  |  |
| 180/1080 | Axelia Paloma Pharma India PVT LTD | Co-Arther | India |  | x |  |  |  |  |  |
| 180/1080 | Baroque Pharma PVT LTD | Ravimal | India |  |  |  | x |  |  |  |
| 180/1080 | Bliss GVS Pharma LTD | Cofantrine | India | x | x |  |  |  |  |  |
| **Strength** | **Manufacturer** | **Brand Name** | **Country of Manufacture** | **Benin** | **DRC** | **Kenya** | **Nigeria** | **Tanzania** | **Uganda** | **Zambia** |
| **Artemether Lumefantrine Suspension *(cont.)*** | | | | | | | | | | |
| 180/1080 | Bliss GVS Pharma LTD | Gvither Plus | India |  | x |  |  |  |  |  |
| 180/1080 | Bliss GVS Pharma LTD | Lonart | India |  | x | x | x | x |  | x |
| 180/1080 | Bliss GVS Pharma LTD | Lufanter | India | x |  |  |  |  |  |  |
| 180/1080 | Caisa Pharma International | Caisther | DRC |  | x |  |  |  |  |  |
| 180/1080 | Cipla LTD | Artemef | India |  |  |  | x |  |  |  |
| 180/1080 | Ciron Drugs & Pharma PVT LTD | Botamil Plus | India |  |  |  | x |  |  |  |
| 180/1080 | Ciron Drugs & Pharma PVT LTD | Ronymal | India |  |  |  | x |  |  |  |
| 180/1080 | Colife | M-2 | DRC |  | x |  |  |  |  |  |
| 180/1080 | Coral Laboratories LTD | Co-Corither | India |  |  | x |  |  |  |  |
| 180/1080 | Cosmos LTD | Lufanate | Kenya |  |  | x |  |  |  |  |
| 180/1080 | Dafra Pharma | Co-Artesiane | Netherlands | x | x | x | x | x | x | x |
| 180/1080 | Doctor Pharma Manufacturing UK LTD | Aludoc | India |  | x |  |  |  |  |  |
| 180/1080 | Emzor Pharma Industries LTD | Lokmal | Nigeria |  |  |  | x |  |  |  |
| 180/1080 | First Swiss Pharma | Nomal | India |  | x |  |  |  |  |  |
| 180/1080 | Fiza Healthcare PVT LTD | Falciart | India | x |  |  |  |  |  |  |
| 180/1080 | Glenmark Pharma LTD | Artiglen Max | India |  |  |  |  |  |  | x |
| 180/1080 | Globela Pharma PVT LTD | Aquamal | India |  |  |  | x |  |  |  |
| 180/1080 | Greenfiled Pharma (Jiangsu) Co. LTD | Atmal | China |  |  |  | x |  |  |  |
| 180/1080 | Greenfiled Pharma (Jiangsu) Co. LTD | Comether | China |  |  |  | x |  |  |  |
| 180/1080 | Jiangsu Ruinian Qianjin Pharma Co LTD | Hanmal | China |  |  |  | x |  |  |  |
| 180/1080 | Jiangsu Ruinian Qianjin Pharma Co LTD | Hatherley | China |  |  |  | x |  |  |  |
| 180/1080 | Jiangsu Ruinian Qianjin Pharma Co LTD | Lomasyl | China |  |  |  | x |  |  |  |
| 180/1080 | Jiangsu Ruinian Qianjin Pharma Co LTD | Micpon | China |  |  |  | x |  |  |  |
| 180/1080 | Jiangsu Ruinian Qianjin Pharma Co LTD | Paluexit | China |  |  |  | x |  |  |  |
| 180/1080 | Jiangsu Ruinian Qianjin Pharma Co LTD | Pemametre | China |  |  |  | x |  |  |  |
| 180/1080 | Jiangsu Ruinian Qianjin Pharma Co LTD | Sarmata | China |  |  |  | x |  |  |  |
| 180/1080 | Jiangsu Ruinian Qianjin Pharma Co LTD | Sivometertrine | China |  |  |  | x |  |  |  |
| 180/1080 | Jiangsu Ruinian Qianjin Pharma Co LTD | Telufan Forte | China |  |  |  | x |  |  |  |
| 180/1080 | Jiangsu Yixing Forward Pharma Factory | Coatal | China |  |  |  | x |  |  |  |
| 180/1080 | Juhel Nigeria LTD | Artemelum | Nigeria |  |  |  | x |  |  |  |
| 180/1080 | Medical Pharma | Bimalaril | China | x |  |  |  |  |  |  |
| 180/1080 | Medinomics Healthcare PVT LTD | Fantem | India | x |  |  |  |  |  |  |
| 180/1080 | Mediwin Pharma | Grutha | India |  |  |  | x |  |  |  |
| 180/1080 | Mekophar Chemical Pharma Joint Stock Co | Artemether Plus | Vietnam |  |  |  | x |  |  |  |
| 180/1080 | Micro Labs LTD | Amatem | India |  |  |  | x |  |  |  |
| 180/1080 | Naxpar Laboratories PVT LTD | Fynale | India |  |  |  | x |  |  |  |
| **Strength** | **Manufacturer** | **Brand Name** | **Country of Manufacture** | **Benin** | **DRC** | **Kenya** | **Nigeria** | **Tanzania** | **Uganda** | **Zambia** |
| **Artemether Lumefantrine Suspension *(cont.)*** | | | | | | | | | | |
| 180/1080 | New Cesamex | Cether-L | DRC |  | x |  |  |  |  |  |
| 180/1080 | Phatkin | Arthefan | DRC |  | x |  |  |  |  |  |
| 180/1080 | Promed | Lumeart | DRC |  | x |  |  |  |  |  |
| 180/1080 | Safi Pharma | Palufin | India |  | x |  |  |  |  |  |
| 180/1080 | Saga Laboratories | Crotan | India |  |  |  | x |  |  |  |
| 180/1080 | Shalina Laboratories PVT LTD | L-Artem | India |  | x |  |  |  |  | x |
| 180/1080 | Shalina Laboratories PVT LTD | Shal’Artem | India |  |  |  | x |  |  |  |
| 180/1080 | Stallion Laboratories PVT LTD | BG Mal | India |  |  |  | x |  |  |  |
| 180/1080 | Swiss Pharma LTD | Arenax Plus | India |  |  |  | x |  |  |  |
| 180/1080 | Swiss Pharma LTD | Menfancil | India |  |  |  | x |  |  |  |
| 180/1080 | Swiss Pharma LTD | Winart | India |  |  |  | x |  |  |  |
| 180/1080 | Tongmei Laboratories | Artome | Togo | x |  |  |  |  |  |  |
| 180/1080 | Vapi Care Pharma PVT LTD | Ogamal | India |  |  |  | x |  |  |  |
| 180/1080 | Sifa Pharma | Arte-Ped | India |  | x |  |  |  |  |  |
| 180/1080 | Yanzhou Xier Kangtai Pharma Co LTD | Malastop | China |  |  |  | x |  |  |  |
| 180/1080 | Yanzhou Xier Kangtai Pharma Co LTD | Voather | China |  |  |  | x |  |  |  |
| 180/1080 | Zenufa Laboratories | Co-Artluf | India |  | x |  |  |  |  |  |
| 180/1080 | Zest Pharma | Luther | India |  | x |  |  |  |  |  |
| 360/2160 | Dafra Pharma | Co-Artesiane | Netherlands | x | x | x | x | x | x | x |
| **Artemether Lumefantrine Other** | | | | | | | | | | |
| 20/120 | Afrab-Chem LTD | La-Tesen Drops | Nigeria |  |  |  | x |  |  |  |
| 20/120 | Jiangsu Ruinian Qianjin Pharma Co LTD | Tamether Granule | China |  |  |  | x |  |  |  |
| 20/120 | Bliss GVS Pharma LTD | Lonart Suppository | India |  |  | x |  |  |  |  |

| **Strength** | **Manufacturer** | **Brand Name** | **Country of Manufacture** | **Benin** | **DRC** | **Kenya** | **Nigeria** | **Tanzania** | **Uganda** | **Zambia** |
| --- | --- | --- | --- | --- | --- | --- | --- | --- | --- | --- |
| **Artemisinin Naphthoquine Tablet** | | | | | | | | | | |
| 125/50 | Kunming Pharmaceutical Co | Arco | China | x | x |  |  | x | x |  |
| 250/100 | Kunming Pharmaceutical Co | Arco | China |  |  | x |  | x |  | x |
| **Artemisinin Piperaquine Tablet** | | | | | | | | | | |
| 62.5/375 | Artepharm Co. LTD | Artequick | China | x | x | x | x |  | x |  |
| 62.5/375 | Watson Global Pharma LTD | Artcop-DS | Nigeria |  |  |  | x |  |  |  |
| **Arterolane Piperaquine Tablet** | | | | | | | | | | |
| 150/750 | Ranbaxy Laboratories LTD | Synriam | India |  |  |  | x | x |  |  |
| **Artesunate Sulfamethoxypyrazine Pyrimethamine Tablet** | | | | | | | | | | |
| 100/250/12.5 | Dafra Pharma | Co-Arinate FDC | Italy | x | x | x | x |  |  | x |
| 100/250/12.5 | Denk Pharma | Asunate Denk | Germany | x | x |  |  |  |  | x |
| 100/250/12.5 | Vardhman Exports | Fansinat Junior | India |  | x |  |  |  |  |  |
| 100/250/12.5 | Zenufa Laboratories | Malacom | DRC |  | x |  |  |  |  |  |
| 50/500/25 | Globela Pharma PVT LTD | Glosunat Plus | India |  |  |  | x |  |  |  |
| 50/500/25 | Plethico Pharma LTD | Artedar | India | x |  |  |  |  |  |  |
| 100/500/25 | Pharmanova LTD | Novidar Plus | Zambia |  |  |  |  |  |  | x |
| 200/500/25 | Alisons SPRL | Falcidox | India |  | x |  |  |  |  |  |
| 200/500/25 | AMT Inc. | Artesunate Plus | DRC |  | x |  |  |  |  |  |
| 200/500/25 | Colife | Arte-Plus | DRC |  | x |  |  |  |  |  |
| 200/500/25 | Dafra Pharma | Co-Arinate FDC | Italy | x | x | x | x |  |  | x |
| 200/500/25 | Denk Pharma | Asunate Denk | Germany | x | x |  |  |  |  | x |
| 200/500/25 | Global Healthcare LTD | Farenax | Nigeria |  |  |  | x |  |  |  |
| 200/500/25 | Micro Labs LTD | Amalar Plus | India |  |  |  | x |  |  |  |
| 200/500/25 | Vapi Care Pharma PVT LTD | Maltarka | India |  |  |  | x |  |  |  |
| 200/500/25 | Zenufa Laboratories | Malacom | DRC |  | x |  |  |  |  |  |

| **Strength** | **Manufacturer** | **Brand Name** | **Country of Manufacture** | **Benin** | **DRC** | **Kenya** | **Nigeria** | **Tanzania** | **Uganda** | **Zambia** |
| --- | --- | --- | --- | --- | --- | --- | --- | --- | --- | --- |
| **Artesunate Amodiaquine Tablet** | | | | | | | | | | |
| 25/75 | Doctor Pharma Manufacturing UK LTD | Armadoc | India |  | x |  |  |  |  |  |
| 50/125 | Group Pharma Michel Iderne | Cospherunat | France | x |  |  |  |  |  |  |
| 50/150 | Doctor Pharma Manufacturing UK LTD | Armadoc | India |  | x |  |  |  |  |  |
| 50/150 | Guilin Pharma Co LTD | Valmonate | China | x |  |  |  |  |  |  |
| 50/153 | Swiss Pharma LTD | Dart | Nigeria |  |  |  | x |  |  |  |
| 50/153 | Vardhman Exports | Amonat Junior | India |  | x |  |  |  |  |  |
| 100/270 | Denk Pharma | ASAQ Denk | Germany | x | x |  |  |  |  |  |
| 100/300 | Adams Pharma Co LTD | Anate | China |  |  |  | x |  |  |  |
| 100/300 | Adams Pharma Co LTD | Artediam | China | x |  |  |  |  |  |  |
| 100/300 | Adams Pharma Co LTD | Camosunate | China |  |  |  | x |  |  |  |
| 100/300 | Adams Pharma Co LTD | Arenax | Belgium |  | x | x |  |  |  |  |
| 100/300 | Doctor Pharma Manufacturing UK LTD | Armadoc | India |  | x |  |  |  |  |  |
| 100/300 | Odypharm LTD | Artediam | China | x |  |  |  |  |  |  |
| 100/306.2 | Shalina Laboratories PVT LTD | Sunat-A | India |  | x |  |  |  |  | x |
| 100/306.2 | Vardhman Exports | Amonat-A | India |  | x |  |  |  |  |  |
| 200/612.4 | Shalina Laboratories PVT LTD | Sunat-A | India |  | x |  |  |  |  |  |
| **Artesunate Amodiaquine Granule** | | | | | | | | | | |
| 25/75 | Adams Pharma Co LTD | Anate | China |  |  |  | x |  |  |  |
| 25/75 | Adams Pharma Co LTD | Camosunate | China |  |  |  | x |  |  |  |
| 25/75 | Unicure | Artesmodia | Nigeria |  |  |  | x |  |  |  |
| 50/150 | Adams Pharma Co LTD | Anate | China |  |  |  | x |  |  |  |
| 50/150 | Adams Pharma Co LTD | Camosunate | China |  |  |  | x |  |  |  |

| **Strength** | **Manufacturer** | **Brand Name** | **Country of Manufacture** | **Benin** | **DRC** | **Kenya** | **Nigeria** | **Tanzania** | **Uganda** | **Zambia** |
| --- | --- | --- | --- | --- | --- | --- | --- | --- | --- | --- |
| **Artesunate Amodiaquine Suspension** | | | | | | | | | | |
| 50/150 | Jiangsu Ruinian Qianjin Pharma Co | MD-Artesunate Plus | China |  |  |  | x |  |  |  |
| 25/50 | Pfizer | Camoquine Plus suspension plus drops | Senegal | x |  |  | x |  |  |  |
| **Artesunate Mefloquine Tablet** | | | | | | | | | | |
| 50/125 | Acino/Mepha | Artequin | Switzerland | x |  | x |  |  |  |  |
| 100/125 | Acino/Mepha | Artequin | Switzerland | x | x | x | x | x | x |  |
| 200/250 | Acino/Mepha | Artequin | Switzerland | x | x | x | x | x | x |  |
| 200/250 | Jiangsu Ruinian Qianjin Pharma Co | Amdin | China |  |  |  | x |  |  |  |
| **Artesunate Mefloquine Granule** | | | | | | | | | | |
| 50/125 | Acino/Mepha | Artequin | Switzerland | x | x | x | x | x | x |  |
| **Dihydroartemisinin Piperaquine Tablet** | | | | | | | | | | |
| 15/120 | GPC | Darte-Q | China | x |  | x |  |  |  |  |
| 20/160 | Beijing Holley-Cotec Pharma LTD | Duo-Cotecxin | China |  |  | x |  | x | x |  |
| 30/225 | Kunimed Pharma LTD | Waipa ACT | Nigeria |  |  |  | x |  |  |  |
| 32/320 | Unicure Pharma LTD | Artexaten | Nigeria |  |  |  | x |  |  |  |
| 40/320 | Ajanta Pharma LTD | Ridmal | India |  |  | x |  | x | x | x |
| 40/320 | Alisons SPRL | Artepip | India |  | x |  |  |  |  |  |
| 40/320 | AMT Inc. | Diperakine | Vietnam |  | x |  |  |  |  |  |
| 40/320 | Award Global Co. LTD | Upxin | India |  |  |  | x |  |  |  |
| 40/320 | Beijing Holley-Cotec Pharma LTD | Arteraquin | China |  |  |  |  | x |  |  |
| 40/320 | Bliss GVS Pharma LTD | P-Alaxin | India | x | x | x | x | x |  |  |
| 40/320 | Bliss GVS Pharma LTD | P-Gvaxin | India |  | x |  |  |  |  |  |
| 40/320 | Coral Laboratories | Duo-Artepep | India |  |  | x |  |  |  |  |
| 40/320 | Dawa LTD | Co-Malasinin | Kenya |  |  | x |  |  |  |  |
| 40/320 | Ga Pharma | Co-Artemax | Greece |  |  |  |  | x |  |  |
| 40/320 | Globela Pharma PVT LTD | Pipart | India |  |  |  | x |  |  |  |
| 40/320 | Globela Pharma PVT LTD | Pymal | India |  |  |  | x |  |  |  |
| 40/320 | GPC | Darte-Q | China | x | x | x |  |  |  |  |
| 40/320 | Greenfiled Pharma (Jiangsu) Co. LTD | Fanmet | China |  |  |  | x |  |  |  |
| 40/320 | Guilin Pharma Co LTD | D-Artepp | China |  | x | x | x | x |  | x |
| 40/320 | Beijing Holley-Cotec Pharma LTD | Duo-Cotecxin | China | x | x | x | x | x | x |  |
| 40/320 | Hubei Meibao Pharma Co LTD | Droa-Quine | China |  |  |  | x |  |  |  |
| 40/320 | Ipca Laboratories LTD | Duoquin | China |  |  |  |  | x |  |  |
| 40/320 | Jiangsu Ruinian Qianjin Pharma Co LTD | Axcin DP | China |  |  |  | x |  |  |  |
| 40/320 | Jiangsu Ruinian Qianjin Pharma Co LTD | Codisin Plus | China |  |  |  | x |  |  |  |
| 40/320 | Laboratorios Salvat S.A | Malacur | India | x | x | x |  | x |  |  |
| **Strength** | **Manufacturer** | **Brand Name** | **Country of Manufacture** | **Benin** | **DRC** | **Kenya** | **Nigeria** | **Tanzania** | **Uganda** | **Zambia** |
| **Dihydroartemisinin Piperaquine Tablet *(cont)*** | | | | | | | | | | |
| 40/320 | Medinomics Healthcare PVT LTD | Dipi | India |  |  |  |  | x |  |  |
| 40/320 | Mekophar Chemical Pharma Joint Stock Co | Falcidin | Vietnam |  |  |  | x |  |  |  |
| 40/320 | Mingshen Pharma Factory | Solartep | China |  |  |  | x |  |  |  |
| 40/320 | OPC Pharma Joint Stock Co | Amnoquine | Vietnam |  |  |  | x |  |  |  |
| 40/320 | Pharbaco Central Pharma | Arterakine | Vietnam |  |  |  | x |  |  |  |
| 40/320 | Pharmagros SPRL | Palucur | India |  | x |  |  |  |  |  |
| 40/320 | Saga Laboratories | Terocan | India |  |  |  | x |  |  |  |
| 40/320 | Shalina Laboratories PVT LTD | Paludose Plus | India |  | x |  |  |  |  |  |
| 40/320 | Universal Corporation LTD | Duotab | Kenya |  |  | x |  |  |  |  |
| 40/320 | Watson Global Pharma LTD | Artcop | Nigeria |  |  |  | x |  |  |  |
| 40/320 | Watson Global Pharma LTD | Arthelad | Nigeria |  |  |  | x |  |  |  |
| 40/320 | Yanzhou Xier Kangtai Pharma Co. LTD | Dipiphate | China |  |  |  | x |  |  |  |
| 40/320 | Yanzhou Xier Kangtai Pharma Co. LTD | Kinotem | China |  |  |  | x |  |  |  |
| 40/320 | Yanzhou Xier Kangtai Pharma Co. LTD | Viskart-P | China |  |  |  | x |  |  |  |
| 40/320 | Zenufa Laboratories | Combiquin | India |  | x |  |  |  |  |  |
| 40/320 | Zhejiang Dongri Pharma Co LTD | Biartem | China |  |  |  | x |  |  |  |
| **Dihydroartemisinin Piperaquine Granule** | | | | | | | | | | |
| 15/120 | GPC | Darte-Q | China | x |  | x |  |  |  |  |
| **Dihydroartemisinin Piperaquine Suspension** | | | | | | | | | | |
| 7.5/60 | Mingshen Pharma. Factory | Solartep | China |  |  |  | x |  |  |  |
| 20/160 | Artepip | Alisons SPRL | India |  | x |  |  |  |  |  |
| 20/160 | AMT Inc. | Diperakine | Vietnam |  | x |  |  |  |  |  |
| 20/160 | Zenufa Laboratories | Combiquin | India |  | x |  |  |  |  |  |
| 80/640 | Bliss GVS Pharma. LTD | P-Alaxin | India | x | x | x | x | x |  |  |
| 80/640 | Bliss GVS Pharma. LTD | P-Gvaxin | India |  | x |  |  |  |  |  |
| 80/640 | Greenfiled Pharma (Jiangsu) Co. LTD | Fanmet | China |  |  |  | x |  |  |  |
| 80/640 | Jiangsu Ruinian Qianjin Pharma Co LTD | Artecxin | China |  |  |  | x |  |  |  |
| 80/640 | Jiangsu Ruinian Qianjin Pharma Co LTD | Axcin DP | China |  |  |  | x |  |  |  |
| 80/640 | Jiangsu Ruinian Qianjin Pharma Co LTD | Codisin Plus | China |  |  |  | x |  |  |  |
| 80/640 | Mekophar Chemical Pharma Joint Stock Co | Falcidin | Vietnam |  |  |  | x |  |  |  |
| 80/640 | Saga Laboratories | Terocan | India |  |  |  | x |  |  |  |
| 80/640 | Yanzhou Xier Kangtai Pharma Co LTD | Viskart-P | China |  |  |  | x |  |  |  |
| 90/720 | Colife | Malaxin-P | DRC |  | x |  |  |  |  |  |
| 90/720 | Laboratorios Salvat S.A. | Malacur | India | x | x | x |  |  |  |  |
| 90/720 | Pharmagros SPRL | Palacur | India |  | x |  |  |  |  |  |
| 240/1920 | Pharbaco Central Pharma | Arterakine | Vietnam |  |  |  | x |  |  |  |

| **Strength** | **Manufacturer** | **Brand Name** | **Country of Manufacture** | **Benin** | **DRC** | **Kenya** | **Nigeria** | **Tanzania** | **Uganda** | **Zambia** |
| --- | --- | --- | --- | --- | --- | --- | --- | --- | --- | --- |
| **Dihydroartemisinin Piperaquine Trimethoprim Tablet** | | | | | | | | | | |
| 16/160/45 | Odypharm LTD | Artecom | China | x |  |  |  |  |  |  |
| 16/160/45 | Tonghe Pharma Co LTD | Artecom | China | x |  |  |  |  |  |  |
| 32/320/90 | Tonghe Pharma Co LTD | Artecom | China | x | x |  |  |  |  |  |
| 32/320/90 | Tonghe Pharma Co LTD | Genaquine | China |  |  |  | x |  |  |  |
| 32/320/90 | Odypharm LTD | Artecom | China | x | x |  |  |  |  |  |
| 32/320/90 | Saga Laboratories | Arthemexin P | India |  |  |  | x |  |  |  |
| **Dihydroartemisinin Piperaquine Trimethoprim Suspension** | | | | | | | | | | |
| 8/80/22.5 | Tiopharms Healthcare Services LTD | Triact | Nigeria |  |  |  | x |  |  |  |
| 10.7/107/30 | Odypharm LTD | Artecom | China | x |  |  |  |  |  |  |
| 10.7/107/30 | Tonghe Pharma Co LTD | Artecom | China | x |  |  |  |  |  |  |
| **Dihydroartemisinin Piperaquine SP Tablet** | | | | | | | | | | |
| 60/500/25 | Bliss GVS Pharma LTD | Alaxin Plus | India | x |  |  |  |  |  |  |
| 160/500/25 | Colife | Malaxin Plus | DRC |  | x |  |  |  |  |  |
