# Supplementary material for: Do anti-malarials in Africa meet quality standards? The market penetration of non quality-assured artemisinin combination therapy in eight African countries
Source: Malar J. 2017 May 25;16:204. doi: 10.1186/s12936-017-1818-8 (PMC5444102; doi:10.1186/s12936-017-1818-8)
Supplement: Supplementary file 2 — Additional file 2. Median private sector price for QAACT and non-QAACT during the most recent survey round (US dollars). [file 12936_2017_1818_MOESM2_ESM.docx]

**Additional File 2: Median private sector price for QAACT and non-QAACT during the most recent survey round (US dollars)**

|  | Benin  2014 | Kinshasa, DRC 2015 | Katanga, DRC  2015 | Nigeria  2015 | Kenya  2014 | Tanzania  2014 | Uganda  2015 | Zambia  2014 |
| --- | --- | --- | --- | --- | --- | --- | --- | --- |
|  | Median  [IQR]^(N)^ | Median  [IQR] ^(N)^ | Median  [IQR] ^(N)^ | Median  [IQR] ^(N)^ | Median  [IQR] ^(N)^ | Median  [IQR] ^(N)^ | Median  [IQR] ^(N)^ | Median  [IQR] ^(N)^ |
| **Tablet formulation price per AETD*** |  |  |  |  |  |  |  |  |
| QAACT | $2.06 | $5.04 | $1.53 | $1.69 | $1.50 | $1.18 | $1.62 | $2.46 |
|  | [1.44-2.89] ^(1802)^ | [0.00-7.40] ^(279)^ | [0.77-2.19] ^(692)^ | [1.27-2.44] ^(8765)^ | [1.13-2.25] ^(2209)^ | [1.18-1.77] ^(3692)^ | [1.13-1.94] ^(5278)^ | [1.64-4.92] ^(265)^ |
| First-line ACT (AL) | $2.06 | $6.58 | $1.75 | $1.69 | $1.50 | $1.18 | $1.55 | $2.46 |
|  | [1.44-2.47] ^(1515)^ | [5.70-9.65]^(137)^ | [1.64-2.63] ^(322)^ | [1.27-2.03] ^(7870)^ | [1.13-2.25] ^(2069)^ | [1.18-1.77] ^(3630)^ | [1.13-1.94] ^(3926)^ | [1.64-4.92] ^(242)^ |
| Other ACT | $5.61 | $0.00 | $1.10 | $2.03 | $1.69 | $1.48 | $1.94 | $6.56 |
|  | [2.06-9.65]^(287)^ | [0.00-2.19] ^(142)^ | [0.44-1.75]^(370)^ | [1.01-3.04]^(895)^ | [1.13-3.38] ^(140)^ | [1.18-2.36] ^(62)^ | [0.97-3.88] ^(1352^) | [1.64-16.08] ^(23)^ |
| Non-QAACT | $7.23 | $3.84 | $4.38 | $3.04 | $4.51 | $6.50 | $3.23 | $5.74 |
|  | [5.12-8.19] ^(4664)^ | [3.07-4.93] ^(4063)^ | [2.19-4.93] ^(1139)^ | [2.37-4.06] ^(4564)^ | [3.38-7.21] ^(2,077)^ | [5.91-8.86] ^(1,864)^ | [1.94-4.85] ^(4946)^ | [3.28-10.17] ^(377)^ |
| First-line ACT (AL) | $5.30 | $3.95 | $4.38 | $3.04 | $3.38 | $3.54 | $1.94 | $4.51 |
|  | [4.77-7.23] ^(2870)^ | [3.29-4.93] ^(2378)^ | [2.19-4.93] ^(775)^ | [2.54-4.06] ^(2816)^ | [2.25-4.34] ^(557)^ | [1.18-4.43] ^(444)^ | [1.55-3.23] ^(1917)^ | [2.63-6.24] ^(222)^ |
| Other ACT | $8.42 | $3.51 | $4.11 | $2.85 | $5.07 | $7.09 | $4.85 | $9.85 |
|  | [7.23-9.63] ^(1794)^ | [2.74-5.32] ^(1685)^ | [3.29-5.48] ^(364)^ | [2.03-3.71] ^(1748)^ | [3.94-10.14] ^(1520)^ | [5.91-9.53] ^(1420)^ | [3.56-5.82] ^(3029)^ | [7.38-16.41] ^(155)^ |
| **Suspension price per bottle** |  |  |  |  |  |  |  |  |
| Non-QAACT | $6.02 | $3.39 | $4.38 | $3.04 | $3.94 | $7.68 | $3.23 | $4.92 |
|  | [4.58-7.23] ^(1621)^ | [2.74-3.84] (^2741)^ | [3.84-4.93] ^(674)^ | [2.44-3.55] ^(1990)^ | [3.38-5.63] ^(923)^ | [6.50-8.86] ^(358)^ | [3.23-4.20] ^(2047)^ | [4.10-6.56] ^(168^) |
| First-line ACT (AL) | $5.30 | $3.29 | $4.38 | $3.04 | $3.94 | $7.68 | $3.23 | $4.92 |
|  | [4.33-7.34] ^(1414)^ | [2.74-3.84] ^(2244)^ | [3.84-4.93] ^(612)^ | [2.54-3.55] ^(1396)^ | [3.60-6.20] ^(651)^ | [6.50-8.86] ^(358)^ | [2.91-4.20] ^(1418)^ | [4.10-6.56] ^(168)^ |
| Other ACT | $7.23 | $3.51 | $4.38 | $2.79 | $3.94 | - | $3.88 | - |
|  | [6.80-7.23] ^(207)^ | [2.74-4.17] ^(497)^ | [3.84-5.48] ^(62)^ | [2.28-3.04] ^(594)^ | [3.38-3.94] ^(272)^ | ^(0)^ | [3.23-4.85] ^(629)^ | ^(0)^ |

The national first line treatment for uncomplicated malaria is AL in Kenya, Tanzania, Uganda and Zambia. Benin, DRC and Nigeria national treatment guidelines indicate AL or Artesunate Amodiaquine. The most commonly available first-line ACT in each country was AL and AL was used for the median first-line price in this table. Madagascar is excluded from this table due to no non-QAACT audited in the private sector.

*AETD - adult equivalent treatment dose - is the number of milligrams of the active ingredient required to treat a 60kg adult. Information provided by the respondent about price for a specific amount of anti-malarial drug (e.g. price per tablet or price per package) was used together with product information to generate price per AETD.
