# Supplementary figures and images for: Do anti-malarials in Africa meet quality standards? The market penetration of non quality-assured artemisinin combination therapy in eight African countries
Source: Malar J. 2017 May 25;16:204. doi: 10.1186/s12936-017-1818-8 (PMC5444102; doi:10.1186/s12936-017-1818-8)

**Additional file 3. Snapshot of market share for non-QAACT across sectors in 8 study countries**


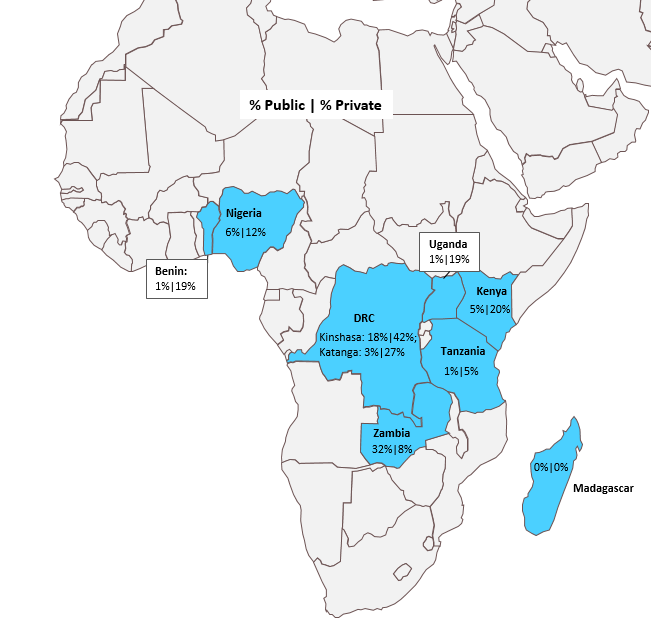

Supplement: Supplementary file 3 — Additional file 3. Snapshot of market share for non-QAACT across sectors in 8 study countries. [file 12936_2017_1818_MOESM3_ESM.docx]
