# Supplementary material for: Do anti-malarials in Africa meet quality standards? The market penetration of non quality-assured artemisinin combination therapy in eight African countries
Source: Malar J. 2017 May 25;16:204. doi: 10.1186/s12936-017-1818-8 (PMC5444102; doi:10.1186/s12936-017-1818-8)
Supplement: Supplementary file 4 — Additional file 4. Non-QAACT anti-malarial market share within each type of private sector outlet. [file 12936_2017_1818_MOESM4_ESM.docx]

**Additional File 4: Non-QAACT anti-malarial market share within each type of private sector outlet**

|  |  | Private For-Profit Health Facility | | Pharmacy | | | | Drug Store | | General Retailer | | Itinerant Drug Vendor | | | Total For-Profit | |
| --- | --- | --- | --- | --- | --- | --- | --- | --- | --- | --- | --- | --- | --- | --- | --- | --- |
|  |  | AETDs (N)* | % Non-QAACT | AETDs (N)* | % Non-QAACT | | | AETDs (N)* | % Non-QAACT | AETDs (N)* | % Non-QAACT | AETDs (N)* | % Non-QAACT | | AETDs (N)* | % Non-QAACT |
| **West & Central Africa** | |  | |  | | | |  | |  | |  | | |  | |
| Benin | 2009 | 743.3 | 2.9% | 1513.5 | | 27.7% | | 0.0 | - | 1536.6 | 0.0% | 336.8 | 0.0% | | 4130.3 | 14.2% |
|  | 2014 | 1048.5 | 8.5% | 7043.6 | | 64.7% | | 84.5 | 19.6% | 6468.8 | 0.2% | 2124.9 | 0.0% | | 16770.2 | 18.7% |
| DRC, Kinshasa | 2009 | 578.7 | 9.3% | 511.5 | | 20.9% | | 4664.7 | 19.5% | 0.0 | - | 0.0 | - | | 5754.9 | 18.6% |
|  | 2013 | 1264.4 | 23.2% | 299.7 | | 66.9% | | 16972.1 | 40.0% | 2.4 | 0.0% | 0.0 | - | | 18538.7 | 39.5% |
|  | 2015 | 827.0 | 30.7% | 45.0 | | 15.0% | | 10707.4 | 42.8% | 0.0 | - | 0.0 | - | | 11579.4 | 42.0% |
| DRC, Katanga | 2013 | 2106.0 | 3.4% | 0.0 | | - | | 13807.7 | 18.3% | 146.1 | 0.1% | 0.0 | - | | 16059.8 | 15.0% |
|  | 2015 | 1989.1 | 16.7% | 235.6 | | 46.6% | | 11494.0 | 27.9% | 0.0 | - | 0.0 | - | | 13718.7 | 26.7% |
| Nigeria | 2009 | 12172.9 | 15.1% | 47224.5 | | 27.0% | | 39993.0 | 4.6% | 1711.4 | 4.3% | 65.1 | 0.0% | | 101166.9 | 5.0% |
|  | 2011 | 2047.0 | 14.1% | 6906.5 | | 20.6% | | 53929.1 | 6.8% | 1564.2 | 2.5% | 532.7 | 0.6% | | 64979.4 | 8.4% |
|  | 2013 | 1763.1 | 10.3% | 9530.8 | | 30.9% | | 34526.0 | 5.3% | 2030.5 | 17.8% | 58.7 | 0.0% | | 47909.1 | 8.0% |
|  | 2015 | 4972.2 | 20.8% | 22957.5 | | 39.7% | | 98878.2 | 9.0% | 1711.5 | 3.3% | 0.0 | - | | 128713.1 | 12.0% |
| **East Africa** | |  | |  | | | |  | |  | |  | | |  | |
| Kenya | 2010 | 4187.7 | 18.4% | 6993.6 | | 24.6% | | 10604.8 | 7.0% | 1682.6 | 0.0% | 3.0 | 0.0% | | 23,471.6 | 10.7% |
|  | 2011 | 5875.2 | 8.8% | 3282.4 | | 10.8% | | 19471.3 | 6.1% | 1340.1 | 3.7% | 0.0 | - | | 29969.1 | 7.0% |
|  | 2014 | 4077.5 | 22.7% | 15788.6 | | 24.6% | | 10777.1 | 14.9% | 1192.9 | 10.7% | 0.0 | - | | 31836.0 | 20.2% |
| Tanzania | 2010 | 211.3 | 8.4% | 1557.6 | | 18.2% | | 4201.6 | 2.9% | 237.2 | 0.0% | 0 | - | | 6207.8 | 3.1% |
|  | 2011 | 2479.6 | 4.2% | 6969.4 | | 15.8% | | 16212.5 | 3.7% | 114.8 | 0.3% | 0 | - | | 25776.2 | 6.1% |
|  | 2014 | 942.4 | 10.5% | 589.9 | | 28.6% | | 11030.4 | 2.7% | 238.6 | 3.9% | 0 | - | | 12801.4 | 5.0% |
| Uganda | 2010 | 5886.3 | 30.4% | 948.6 | | 52.6% | | 7859.33 | 19.3% | 63.4 | 1.2% | 16.9 | 71.0% | | 14774.6 | 24.9% |
|  | 2011 | 17718.1 | 28.6% | 7026.0 | | 25.5% | | 12483.8 | 10.0% | 52.1 | 0.0% | 0.0 | | - | 37280.0 | 20.4% |
|  | 2013 | 8579.3 | 20.2% | 7054.8 | | 19.8% | | 12,023.3 | 6.2% | 0.0 | - | 0.0 | | - | 27,657.4 | 13.0% |
|  | 2015 | 16760.3 | 22.4% | 10032.6 | | 30.3% | | 22,465.9 | 13.7% | 0.0 | - | 0.0 | | - | 49,258.9 | 18.6% |
| **Southern Africa** | |  | |  | | | |  | |  | |  | | |  | |
| Madagascar | 2010 | 1091.5 | 0.3% | 3659.5 | | | 2.2% | 1110.5 | 0.0% | 2870.0 | 0.0% | 0.0 | | - | 8731.5 | 0.2% |
|  | 2011 | 228.8 | 0.0% | 2286.0 | | | 0.7% | 1733.7 | 0.0% | 1918.0 | 0.0% | 0.0 | | - | 6166.5 | 0.1% |
|  | 2013 | 738.3 | 3.0% | 2501.6 | | | 0.7% | 987.0 | 0.0% | 490.4 | 0.0% | 170.3 | | 0.0% | 4887.5 | 0.5% |
|  | 2015 | 821.9 | 0.0% | 3030.4 | | | 0.0% | 1188.7 | 0.0% | 364.9 | 0.0% | 0.0 | | - | 5405.9 | 0.0% |
| Zambia | 2009 | 1112.5 | 5.8% | 1454.4 | | | 16.7% | 2310.6 | 0.1% | 373.4 | 0.0% | 13.0 | | 0.0% | 5263.9 | 4.9% |
|  | 2011 | 934.1 | 49.2% | 2329.9 | | | 36.0% | 3512.0 | 1.7% | 446.0 | 0.0% | 0.0 | | - | 7220.6 | 19.0% |
|  | 2014 | 367.4 | 33.3% | 687.5 | | | 34.9% | 2041.6 | 2.1% | 383.3 | 0.3% | 0.0 | | - | 3479.9 | 8.1% |
| * Un-weighted number of AETDs distributed within the outlet type for the specified survey year. The number of AETDs distributed is dependent on the sample size and is therefore not tied to total anti-malarial market volume. Total numbers of AETDs distributed should therefore not be compared within a country across survey rounds or between countries and interpreted as indicative of differences in total anti-malarial market size. | | | | | | | | | | | | | | | | |
