# Supplementary material for: Do anti-malarials in Africa meet quality standards? The market penetration of non quality-assured artemisinin combination therapy in eight African countries
Source: Malar J. 2017 May 25;16:204. doi: 10.1186/s12936-017-1818-8 (PMC5444102; doi:10.1186/s12936-017-1818-8)
Supplement: Supplementary file 5 — Additional file 5. Urban rural and private sector outlet type market share for all anti-malarials. [file 12936_2017_1818_MOESM5_ESM.docx]

**Additional file 5: Urban rural and private sector outlet type market share for all anti-malarials**

Notes: Madagascar was not included as there were no quality-assured ACTs in the private sector in 2015 in Madagascar
